# Integrating single-cell multimodal epigenomic data using 1D convolutional neural networks

**DOI:** 10.1093/bioinformatics/btae705

**Published:** 2025-01-16

**Authors:** Chao Gao, Joshua D Welch

**Affiliations:** Department of Computational Medicine and Bioinformatics, University of Michigan, Ann Arbor, MI 48109, United States; Department of Computational Medicine and Bioinformatics, University of Michigan, Ann Arbor, MI 48109, United States; Department of Computer Science and Engineering, University of Michigan, Ann Arbor, MI 48109, United States

## Abstract

**Motivation:**

Recent experimental developments enable single-cell multimodal epigenomic profiling, which measures multiple histone modifications and chromatin accessibility within the same cell. Such parallel measurements provide exciting new opportunities to investigate how epigenomic modalities vary together across cell types and states. A pivotal step in using these types of data is integrating the epigenomic modalities to learn a unified representation of each cell, but existing approaches are not designed to model the unique nature of this data type. Our key insight is to model single-cell multimodal epigenome data as a multichannel sequential signal.

**Results:**

We developed ConvNet-VAEs, a novel framework that uses one-dimensional (1D) convolutional variational autoencoders (VAEs) for single-cell multimodal epigenomic data integration. We evaluated ConvNet-VAEs on nano-CUT&Tag and single-cell nanobody-tethered transposition followed by sequencing data generated from juvenile mouse brain and human bone marrow. We found that ConvNet-VAEs can perform dimension reduction and batch correction better than previous architectures while using significantly fewer parameters. Furthermore, the performance gap between convolutional and fully connected architectures increases with the number of modalities, and deeper convolutional architectures can increase the performance, while the performance degrades for deeper fully connected architectures. Our results indicate that convolutional autoencoders are a promising method for integrating current and future single-cell multimodal epigenomic datasets.

**Availability and implementation:**

The source code of VAE models and a demo in Jupyter notebook are available at https://github.com/welch-lab/ConvNetVAE

## Graphical abstract

**Figure:**
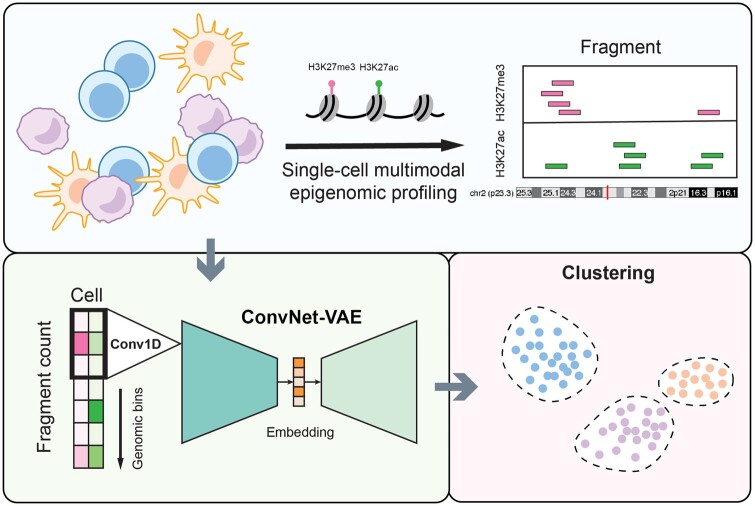

## 1 Introduction

Single-cell sequencing technologies have revolutionized our understanding of cellular heterogeneity and the complexity of biological systems. Recently, single-cell multimodal chromatin profiling has emerged as an exciting new experimental approach to investigate the cellular epigenetic landscape. Two independent studies fused nanobodies (nb) to a transposase enzyme (Tn5) and used these nb–Tn5 conjugates to detect up to three epigenome layers (histone modification or chromatin accessibility) within the same cell (Bartosovic and Castelo-Branco 2022, Stuart *et al.* 2023). The nano-CUT&Tag (nano-CT) and single-cell nanobody-tethered transposition followed by sequencing (scNTT-seq) technologies can, in principle, be used to detect transcription factor binding as well, though this has not yet been demonstrated. These multimodal datasets provide simultaneous measurements of multiple epigenomic layers within individual cells, offering unprecedented opportunities to unravel how histone modifications and chromatin states drive cellular diversity. For example, one can use these types of data to investigate how different histone modifications at the same genomic locus combine to activate or repress transcription of nearby genes. However, the structure of single-cell multimodal epigenomic data is unique compared to other single-cell data types: each modality is a 1D genomic track, and the total measurement for a cell consists of multiple 1D tracks measured at the same genomic positions. This is quite different from any other type of single-cell data—such as single-cell RNA sequencing (scRNA-seq), single-nucleus assay for transposase accessible chromatin with high-throughput sequencing (snATAC-seq), cellular indexing of transcriptomes and epitopes by sequencing (CITE-seq), or 10x Genomics Single Cell Multiome—in which the space of features is most naturally represented in terms of genes or discrete peaks.

A number of computational approaches have been designed to perform joint dimension reduction on single-cell multimodal data types such as CITE-seq and 10x Genomics Single Cell Multiome. For example, the Seurat weighted-nearest neighbor (WNN) algorithm, the multiomic factor analysis (MOFA+), and the MultiVI perform linear or nonlinear dimension reduction on single-cell multimodal datasets that can be represented as genes and peaks (Argelaguet *et al.* 2020, Hao *et al.* 2021, Ashuach *et al.* 2023). Approaches based on variational autoencoders (VAEs) are especially powerful for learning joint representations from single-cell multimodal data. VAEs are unsupervised probabilistic deep learning models that excel at distilling compact and meaningful representations of complex data, as evidenced by their successful applications in scRNA-seq data integration (Lopez *et al.* 2018). For multimodal problems, a VAE based on the concept of the product of experts (PoE) was introduced (Wu and Goodman 2018). This method factorizes the joint distribution over the latent variables into a product of conditional distributions, each representing the output of a modality-specific “expert” model. Each expert comprises an encoder and a decoder, designed to model a specific data modality. Beyond their initial applications in image transformation and machine translation, such VAEs have been adapted for multimodal single-cell sequencing data. For example, Multigrate applies PoE to integrate paired measurements, such as gene expression and chromatin accessibility (peaks), and learn a unified cell embedding for cell clustering and visualization (Lotfollahi *et al.* 2022). Although multimodal VAEs can use any type of neural network layers, single-cell multimodal VAEs have only used fully connected layers due to the unordered nature of gene features. Thus, we refer to these previous approaches as FC-VAEs.

However, directly applying such approaches to single-cell multimodal epigenomic data has several disadvantages. First, by using a peak-centric feature representation, previous approaches neglect the ordered sequential nature of single-cell epigenomic data, in which the epigenomic state of a particular locus shares strong conditional dependence with the states of loci immediately before and after it in linear genome order. Next, the existing approaches treat the features from each epigenomic track independently, ignoring the multitrack nature of single-cell multimodal epigenomic data, removing the crucial information of shared genome position across modalities. This second limitation is especially problematic because it prevents integration algorithms from learning the relationship among different epigenome modalities at a given position within a single cell, which is one of the key motivations for performing single-cell multimodal epigenomic measurement in the first place. To tackle this challenge with a peak-centric approach, the peak locations must be merged, since the locations of peaks typically vary across different modalities (such as H3K27ac versus H3K27me3). Using genomic bins with fixed locations avoids this need for merging peak locations.

1D convolutional neural networks (1D CNNs) have shown success in the analysis of sequential data, especially when the spatial or temporal relationships within the data are crucial (Kiranyaz *et al.* 2021). In particular, deep learning models using 1D convolutional layers have been widely used in the analysis of bulk RNA-seq and bulk epigenome data. Such networks have been trained on bulk data from cell lines and tissues to predict transcriptional and epigenetic profiles from DNA sequence (Kelley *et al.* 2018, Chen *et al.* 2022). Recently, Yuan and Kelley (2022) extended this line of work to single-cell ATAC-seq data: scBasset takes DNA sequences as input and utilizes CNNs to predict chromatin accessibility in single cells. However, to our knowledge, only FC-VAEs have been used to perform dimension reduction and integration of single-cell data.

Here, we present a novel 1D convolutional variational autoencoder framework (ConvNet-VAEs) tailored for integrating single-cell multimodal epigenomic data. We model single-cell multimodal epigenomic data as a multichannel sequential signal. A key innovation of our method is that, by performing convolution over ordered feature space, it adopts a more appropriate inference bias than VAEs with only the fully connected layers that are suitable for unordered features. Our approach combines two streams of work: 1D CNNs for bulk genomic data and VAEs for dimension reduction of single-cell data. Importantly, our method is fundamentally different from this previous work in several key aspects: (i) we utilize a window-based genome binning strategy on the multimodal profiles from single cells and model the fragment count in each bin; (ii) we use 1D convolutional layers that operate over different epigenetic modalities instead of nucleotide bases; and (iii) unlike the previous multimodal VAEs, ConvNet-VAEs consist of only one encoder–decoder pair. We show that ConvNet-VAEs can leverage the strengths of both VAEs and convolution. They effectively reduce data dimensionality and extract local genomic features with a more economical parameter usage compared to that of FC-VAEs.

## 2 Materials and methods

### 2.1 Generative probabilistic model of epigenomic data

We model the count data for a given feature (e.g. a histone modification such as H3K27ac) using a Poisson distribution. Consider multimodal single-cell data consisting of *M* modalities from *B* different experimental batches, with a total of *N* cells. All modalities share the same set of features *G* (e.g. binned genomic regions). We represent cell *i* with a latent factor $z^{i}$ sampled from N(0,I), characterized by batch information $b^{i}$, and a modality-specific library size factor $l_{m}^{i}$. We model the generative process of the count $x_{mg}^{i}$ of the molecular feature *g* within modality *m* (m∈{1,2,…,M}) as follows:

(1) $$\begin{array}{cc} \rho_{mg}^{i} & =f^{\mathrm{Dec}}(z^{i},b^{i})\\ w_{mg}^{i} & =\text{softmax}(\rho_{mg}^{i})\\ \lambda_{mg}^{i} & =w_{mg}^{i}l_{m}^{i}\\ x_{mg}^{i} & ∼\text{Poisson}(\lambda_{mg}^{i}) \end{array}$$

Here, $x_{mg}^{i}\in N_{0}$ represents the count data, $z^{i}\in R^{D}$ is the latent representation of each cell in a *D*-dimensional space, with *D* selected according to the complexity of the data. The modality-specific library size factor is denoted as $l_{m}^{i}\in N_{0}$. $b^{i}$ is a *B*-dimensional one-hot encoded vector containing batch information. The function *f*^Dec^ denotes the decoder neural network, which consists of convolutional layers and/or fully connected (FC) layers. Through the application of a softmax activation function in the final layer, the decoder network maps the latent factors and batch label of cell *i* to the original feature space. In this study, we also implemented negative binomial (NB) distribution in the models, which is able to accommodate overdispersion in the data by including an extra parameter for dispersion. For each gene, the same dispersion parameter of NB is shared across all cells. In our experiments spanning three single-cell multimodal datasets, we observed that ConvNet-VAEs employing NB distribution exhibited negligible differences compared to those utilizing Poisson distributions (Supplementary Fig. S6). Under this distributional assumption, ConvNet-VAEs also maintain their edge over FC-VAEs (Supplementary Figs S7–S9). With these observations, our studies focus on Poisson-based modeling in the rest of this report.

### 2.2 Multimodal VAEs

#### 2.2.1 Variational autoencoders

As previously described, we consider the observed feature vector $x_{m}$ of a cell derived from hidden variable **z**, from batch **b**. Researchers have harnessed the VAE framework for efficient approximation of the posterior distribution for **z** (Kingma and Welling 2013). VAEs, as deep generative models, exploit neural networks for variational inference, facilitating representation learning from high-dimensional data. The functionality is crucial for single-cell data integration and subsequent cell type identification (Lopez *et al.* 2018). Typically, VAEs are trained to optimize the evidence lower bound (ELBO) using stochastic gradient methods. In a unimodal scenario where *M* = 1, the ELBO for a feature vector $x_{1}$ is defined as follows:

(2) $$\begin{array}{c} \text{ELBO}(x_{1})≜E_{q_{\phi }(z|x_{1},b)}[\text{log}\;p_{\theta }(x_{1}|z,b)]\\ -D_{KL}(q_{\phi }(z|x_{1},b)\|p(z), \end{array}$$

where $q_{\phi }(z|x_{1},b)$ and $p_{\theta }(x_{i}|z,b)p(z)$ are the inference model (parameterized by ϕ) and generative model (parameterized by *θ*), respectively. Parameters ϕ and *θ* are optimized by training the neural networks. Throughout this study, we let the posterior of **z** be a multivariate Gaussian with a diagonal covariance structure. To address the challenges in modeling multimodal single-cell data, we introduce multimodal VAEs in the next sections.

#### 2.2.2 Convolutional VAEs with 1D convolutional layers (ConvNet-VAE)

Convolutional neural networks (CNNs) effectively perform tasks such as data compression and classification by learning representations of the input (i.e. 1D for signals or sequences, 2D for images) (LeCun *et al.* 2015). In the context of 1D CNNs, Conv1D filters work on the 1D input sequences and move in one direction. We introduce ConvNet-VAE, a VAE architecture that utilizes 1D convolutional layers to model and integrate single-cell multimodal epigenomic data. By incorporating Conv1D layers, ConvNet-VAE efficiently embeds high-dimensional multimodal epigenomic features of the cells into a low-dimensional space suitable for clustering tasks. For compatibility with 1D CNN, we treat the fragment count of different modalities along the binned genome as 1D sequence with multiple channels, where each channel corresponds to a different modality. Given *N* cells, then we have ${\left\{X^{i}\right\}}_{i=1}^{N}$, and $X^{i}\in N_{0}^{M\times G}$. For instance, in a bi-modal setting, $x_{1\cdot }^{i}$ denotes the first channel, and $x_{2\cdot }^{i}$ the second. The ELBO is formulated as below:

(3) $$\text{ELBO}(X)≜E_{q_{\phi }(z|X,b)}[\text{log}\;p_{\theta }(X|z,b)]-D_{KL}(q_{\phi }(z|X,b)||p(z).$$

Note that we assume that different modalities $x_{m}$ (channels of **X**) are conditionally independent on **z** and **b** for tasks involving multiple modalities.

**Figure 1.**
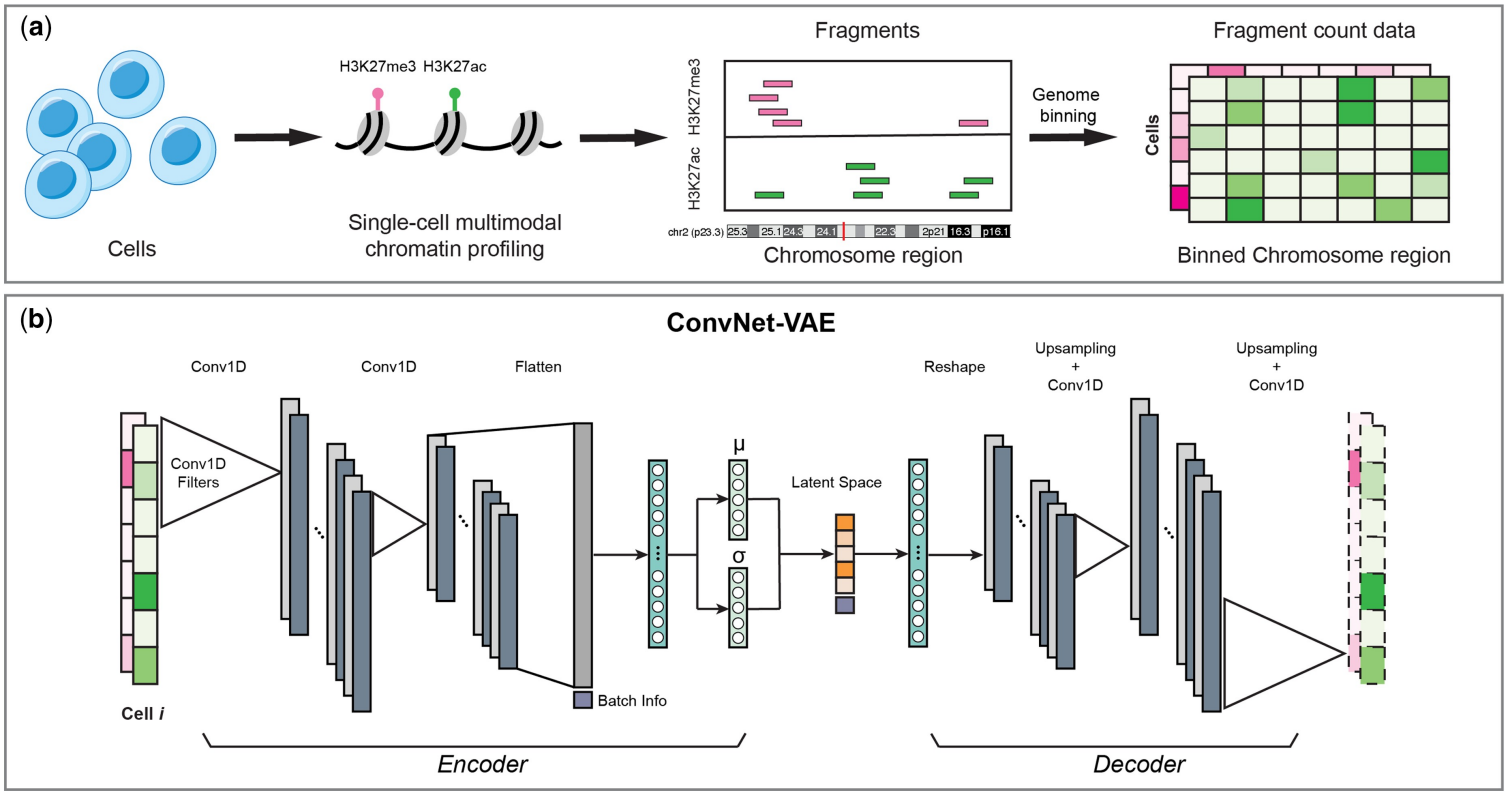
Overview of ConvNet-VAE. (a) For each cell, the fragments of each measured epigenomic modality are acquired by multimodal single-cell epigenome profiling (e.g. H3K27ac + H3K27me3). Followed by genome binning, we obtain the fragment count data with dimension cell × modality × bin. (b) ConvNet-VAE applies 1D convolution and learns low-dimensional representations of the cells from the binned multimodal fragment count of input.

**Figure 2.**
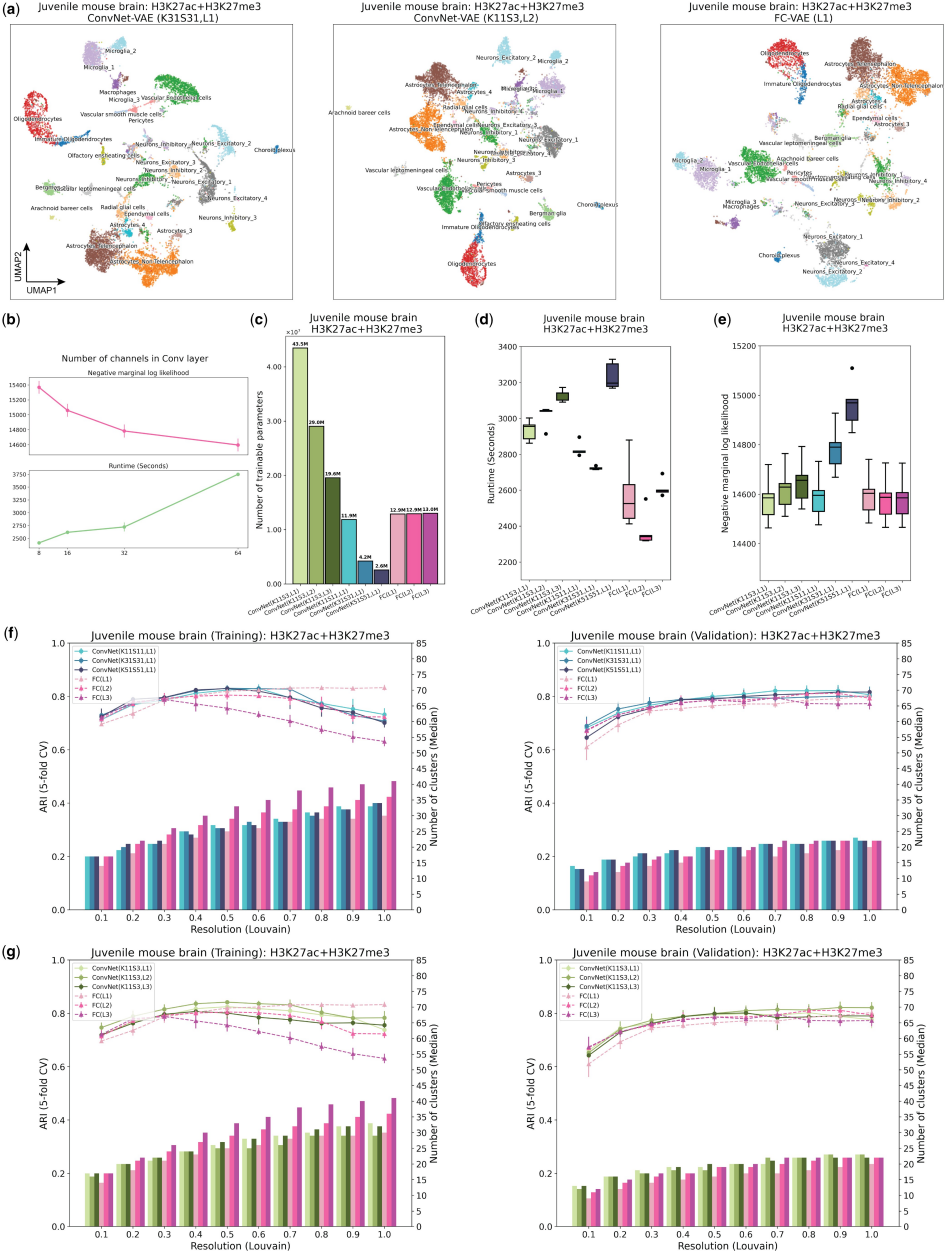
ConvNet-VAEs integrate single-cell bi-modal epigenomic profiling data from mouse brain. (a) UMAP visualization of cell embeddings from ConvNet-VAEs (left, middle) and FC-VAE (right). (b) For single-Conv1D-layer ConvNet-VAE, more channels in the convolutional layer lead to a larger marginal log-likelihood of the validation set at the cost of longer runtime in training, according to the result from five-fold cross-validation. (c) The number of trainable parameters depends on the number of Conv1D layers and stride. ConvNet-VAEs from Group 1 require fewer parameters than FC-VAEs while those from Group 2 need more parameters. (d) Training time across five-fold cross-validation is reported for each model. (e) Negative marginal log-likelihood of validation set estimated through importance sampling (five-fold cross-validation). A lower value implies a larger marginal log-likelihood. (f) Comparisons between ConvNet-VAEs with single Conv1D layer (Group 1) and FC-VAEs in terms of the quality of cell embeddings (training set: left; validation set: right). The bars show the median number of clusters obtained by the Louvain algorithm from five splits in cross-validation over a range of resolutions. The corresponding average Adjusted Rand Index (ARI) is calculated by comparing the resulting clusters to the published cell type labels, displayed as a line plot. Error bars indicate SD across five-fold cross-validation. (g) Comparisons between ConvNet-VAEs with multiple Conv1D layer (Group 2) and FC-VAEs in terms of cell embeddings’ quality (training set: left; validation set: right), exhibited in the same way as (f).

The architecture of ConvNet-VAE is depicted in Fig. 1. This research focuses on two main configurations of ConvNet-VAE models. The first group of models comprises a single convolutional layer with varying sizes of kernel (K) and stride (S). The second group features multiple convolutional layers with constant kernel size and stride. By experimenting single-Conv1D-layer ConvNet-VAE (with a kernel size of 31 and stride of S31) on the bi-modal juvenile mouse brain dataset, we notice an increase in the marginal log-likelihood of validation data when more kernels (output channels) are applied. However, there is a disproportionately large increase in computational time compared to the gains in capturing the data distribution when the kernel count is doubled from 32 to 64 (Fig. 2b). Therefore, for single-Conv1D-layer ConvNet-VAEs, we set the kernel count to 32. In the case of models incorporating a second or third convolutional layer, the output channels are set to 64 and 128, as is commonly done in CNN architectures. Complete specifications are provided in Table 1, including the number of feature channels produced by the convolutional layers (indicated in the parentheses). The final Conv1D layer in the decoder produces an output with a channel count that matches the number of data modalities.

**Table 1.** ConvNet-VAE model architecture.

Grp.	Kernel (K)	Stride (S)	Encoder layers					Decoder layers			
1	11	11	Conv1D(32)			FC	FC(*μ*,*σ*)	FC			Conv1D
	31	31	Conv1D(32)			FC	FC(*μ*,*σ*)	FC			Conv1D
	51	51	Conv1D(32)			FC	FC(*μ*,*σ*)	FC			Conv1D
2	11	3	Conv1D(32)			FC	FC(*μ*,*σ*)	FC			Conv1D
	11	3	Conv1D(32)	Conv1D(64)		FC	FC(*μ*,*σ*)	FC		Conv1D(32)	Conv1D
	11	3	Conv1D(32)	Conv1D(64)	Conv1D(128)	FC	FC(*μ*,*σ*)	FC	Conv1D(64)	Conv1D(32)	Conv1D

Two groups of ConvNet-VAEs are employed in this study. In Group 1, the encoders and decoders of ConvNet-VAEs comprise only one 1D convolutional layer, with increasing size of the kernel (K) and stride (S) of filters (from 11 to 51). In the second group, the ConvNet-VAEs contain an increasing number of 1D convolutional layers (from 1 to 3), with fixed kernel size and stride.

In general, Conv1D and FC layers are followed by Batch Normalization (1D), ReLU activation, and Dropout layers. We perform softmax activation on the output from the last decoding Conv1D layer, without any other transformation. FC(*μ*,*σ*), as well as the FC layer in the decoder, are linear layers. The pooling layer is replaced by applying a large stride (≥3). For enhanced numerical stability, each input channel—representing a different modality—undergoes log transformation [log(x+1)].

#### 2.2.3 VAEs with FC layers (FC-VAE)

In order to demonstrate the advantage of ConvNet-VAE, we include FC-VAE for benchmark analyses. To address the problem of learning joint representations of multiple modalities, the idea of PoE was introduced by Wu and Goodman (2018). We adapted the PoE approach for our specific task of multimodal inference within the context of single-cell epigenomics. Consistent with the settings described in the previous sections, we establish the following joint posterior by assuming conditional independence between $p(x_{m}|z,b)$,

(4) $$p(z|X,b)=p(z|x_{1},x_{2},\ldots ,x_{M},b)\propto \frac{\prod_{m=1}^{M}p(z|x_{m},b)}{\prod_{m=1}^{M-1}p(z)}.$$

We further approximate the true single-modality posterior $p(z|x_{m},b)$ using $q(z|x_{m},b)$ (a Gaussian “expert”) learned from modality-specific neural networks (parameterized by $\phi_{m}$).

(5) $$q(z|x_{m},b)\equiv q_{\phi_{m}}(z|x_{m},b)p(z).$$

The product of Gaussian experts is still Gaussian distributed (Cao and Fleet 2014). Assuming that the *m*th expert outputs *μ_m_* and *V_m_* and setting $T_{m}\equiv V_{m}^{-1}$, we can define the product Gaussian of **z** with the following parameters:

(6) $$\begin{array}{c} \mu_{\mathrm{PoE}}=\frac{\sum_{m}\mu_{m}T_{m}}{{\left(\sum_{m}T_{m}\right)}^{-1}},\\ \Sigma_{\mathrm{PoE}}={\left(\sum_{m}T_{m}\right)}^{-1},\\ \text{ELBO}(x_{1},\ldots ,x_{M})≜E_{q_{\phi }(z|x_{1},\ldots ,x_{M},b)}[\sum_{x_{m}\in X}\;\text{log}\;p_{\theta }(x_{m}|z,b)]\\ -D_{KL}(q_{\phi }(z|x_{1},\ldots ,x_{M},b)||p(z)]). \end{array}$$

We configured FC-VAEs under three different settings with their architectures detailed in Table 2. Each expert model is comprised of two FC layers in the encoder and an additional two FC layers in the decoder, similar to that employed in scVI (Lopez *et al.* 2018). An example model architecture is shown in Supplementary Fig. S1. FC-VAEs apply Batch Normalization (1D), ReLU activation, and Dropout layers in the FC layers, except for FC(*μ*,*σ*) and the final FC decoding layer. The models are trained to optimize the ELBO defined as Equation (6). In addition to the comparable architectures of FC-VAEs and ConvNet-VAEs, we use exactly the same dimension (*D*) for the latent space, dropout rate, training/validation data splits, training scheme, and parameters for clustering, to ensure fair comparison (detailed in Section 2.7).

**Table 2.** FC -VAE model architecture.

	Layers^a^	Encoder layers				Decoder layers			
For each expert	1	FC			FC(*μ*,*σ*)			FC	FC
	2	FC	FC		FC(*μ*,*σ*)		FC	FC	FC
	3	FC	FC	FC	FC(*μ*,*σ*)	FC	FC	FC	FC

For FC-VAEs based on product of experts, the number of layers in each expert (modality-specific encoder–decoder) differ across each FC-VAE variant. Taking FC-VAE (L1) as an example, for each expert, the encoder contains one fully connected layer in addition to one that outputs mean and log-variance parameters, and the decoder consists of two fully connected layers.

a Number of encoding layers [excluding FC(*μ*,*σ*)].

### 2.3 Evaluation on multimodal data integration

We evaluated the efficacy of ConvNet-VAEs in data integration against several unsupervised models, which include the baseline model FC-VAEs, the published Multigrate (Lotfollahi *et al.* 2022) and MOFA+ (Argelaguet *et al*. 2020) frameworks. We implemented the ConvNet-VAEs, FC-VAEs, and MOFA+ with the Poisson modeling assumption. For Multigrate, we used log normalized fragment counts of the epigenomic features as the input and mean squared error as the loss, as suggested by its tutorial. We did not include MultiVI as it was designed for gene expression, ATAC, and potentially protein measurement as the third modality. It cannot integrate multimodal epigenomic data without significant modification to the source code.

We selected average silhouette width (ASW) (Batch), graph connectivity, graph iLISI, and kBET to assess batch-effect removal, and ASW (Cell type), NMI and graph cLISI to check biological conservation. These metrics were introduced by Luecken et al. (2022) for benchmarking single-cell data integration. ASW quantifies the separation of clusters. ASW (Batch) measures batch mixing, ranging from 0 to 1, where 1 indicates perfect mixing. Graph connectivity investigates how well the cells with the same identity are connected in the *k*-nearest neighbor (*k*NN) graph built from integrated data. A graph connectivity score of 1 implies good integration, where all cells with the same label are connected in the *k*NN graph. Korsunsky *et al.* (2019) employed integration Local Inverse Simpson’s Index (iLISI) to measure the batch distribution using local neighbors chosen on a predefined perplexity. As an extension, Graph iLISI can take graph-based integration outputs and a higher score represents better data integration. *k*-NN batch-effect test, known as kBET, starts by constructing a *k*NN graph, and then examines the batch label distribution in the cell’s neighborhood against the global batch label distribution through random sampling (Büttner *et al.* 2019). To investigate the biological conservation, ASW (Cell type) follows the classical definition of ASW using cell type labels. Normalized mutual information (NMI) evaluates overlap between cell-type labels and Louvain clusters calculated from the integrated dataset. Graph cLISI, also based off LISI, measures the separation of cell clusters. The detailed descriptions of these metrics are available in their original publications. For all of them, higher score indicates better performance. We also reported an overall score by aggregating the batch-effect removal and biological conservation scores following the same formula described in the previous benchmark study (Luecken *et al.* 2022).

In this benchmark analysis, we trained VAE models using the entire dataset and 5 different random initializations. We computed these metrics with default settings using the resulting cell embeddings. All metrics reported in this study are average scores across 5 runs.

### 2.4 Evaluation of VAEs’ ability to capture data distribution

To benchmark Bayesian probabilistic models in a uni-modal setting ($x_{1}$), a popular strategy is to compare the marginal likelihood. A VAE model that is better at capturing the data distribution and generating samples is expected to achieve a higher marginal log-likelihood $\text{log}\;p(x_{1})$ on the test set. Similarly, here we used joint conditional log-likelihood $\text{log}\;p(x_{1},x_{2})$ and $\text{log}\;p(x_{1},x_{2},x_{3})$ as the evaluation metrics in the multimodal settings, to compare the quality of the tested deep generative models. These marginal log-likelihoods (marginal with respect to latent variable **z**) can be approximated through importance sampling (Owen and Zhou 2000, Wu and Goodman 2018). Assuming test data $x_{1},\;x_{2}$, as well as the latent representation **z** from a given sample *i* in the bi-modal setting, we have

(7) $$\text{log}\;p(x_{1},x_{2}|b)\approx \;\text{log}\;E_{q_{\phi }(z|x_{1},x_{2},b)}\left[\frac{p_{\theta }(x_{1},x_{2}|z,b)p(z)}{q_{\phi }(z|x_{1},x_{2},b)}\right].$$

The right-hand side of Equation (7) can be estimated by Equation (8):

(8) $$\begin{array}{c} \;\text{log}\;E_{q_{\phi }(z|x_{1},x_{2},b)}\left[\frac{p_{\theta }(x_{1},x_{2}|z)p(z,b)}{q_{\phi }(z|x_{1},x_{2},b)}\right]\\ \approx \;\text{log}\;\frac{1}{N_{S}}\sum_{s=1}^{N_{s}}\frac{p_{\theta }(x_{1}|z_{s},b)p_{\theta }(x_{2}|z_{s},b)p_{N(0,1)}(z_{s})}{q_{N(\mu (x_{1},x_{2},b),\sigma (x_{1},x_{2},b))}(z_{s})}, \end{array}$$

where the samples *z_s_* are randomly drawn from the importance distribution $N(\mu (x_{1},x_{2},b),\sigma (x_{1},x_{2},b))$ defined by the output from the inference networks. *N_s_* is the number of importance samples. $p_{\theta }(x_{1}|z_{s})$ and $p_{\theta }(x_{2}|z_{s})$ are calculated with data distribution obtained by the decoders. We estimated the mean joint log-likelihood of the validation set of 100 importance samples (*N_s_* = 100) on all datasets and reported the average values over five-fold cross-validation.

### 2.5 Evaluation of the cell representations learned by VAEs

We applied the Louvain community detection algorithm (Waltman and Van Ec 2013) to the low-dimensional representations of cells generated by the models on the training and validation sets. Resolution is a parameter that influences the number of identified clusters—a higher value yields more clusters. By running the Louvain clustering over a range of resolution values, we then compare the resulting clusters against the published cell type annotations using the Adjusted Rand Index (ARI) (Hubert and Arabie 1985) as a measure of how well the learned representations capture the underlying structure of the data.

### 2.6 Data preprocessing

#### 2.6.1 Juvenile mouse brain

Bartosovic and Castelo-Branco (2022) recently developed nano-CT technology, enabling multimodal chromatin profiling at single-cell resolution. The authors succeeded in measuring up to three modalities—ATAC, H3K27ac, and H3K27me3—simultaneously within individual cells from the mouse brains (19-day old). Starting with the fragment data of each modality, we used Signac to segment the genome into windows, resulting in a count matrix (fragment count in each genomic bin) with the dimension of cell by bin (Stuart *et al.* 2021). Fang *et al.* (2021) showed that bin size ranging from 1 to 10 kb performed similarly in their benchmark studies. Therefore, we set the bin width to be 10 kb to reduce the input dimension for this analysis. We excluded the bins that overlap with the regions in the ENCODE mouse genome (mm10) blacklist (Amemiya *et al.* 2019). We retained the cells with the authors’ annotation for the analysis. After filtering, H3K27ac and H3k27me3 were measured in total *n* = 11 981 cells (four biological replicates: $n_{1}=2117,\;n_{2}=2479,\;n_{3}=2392,\;n_{4}=4993$), and 4434 of them (two biological replicates: $n_{1}=2084,\;n_{3}=2350$) have additional ATAC measurements. We further selected the 25 000 bins with the largest counts jointly from all of the modalities (i.e. the union of bins of the highest fragment count of each modality) to reduce sparsity. We also explored the model performance using tri-modal data without any bin selection, where there are a total of 245 220 bins. The result indicates that using all of the bins does not significantly improve the model performance (*P* = .064, one-sided *t*-test) (Supplementary Fig. S2), yet it requires much more memory usage (∼4×) and longer runtime (∼10×). For bi-modality data, we used 28 cell type labels generated on the H3K27ac (similar to labels generated on H3K27me3) for model performance evaluation. For the dataset encompassing three modalities, we utilized 26 cell classes from the WNN analysis conducted by the authors. For CNNs, we constructed 3D input arrays (cell × modality × bin).

#### 2.6.2 Human bone marrow mononuclear cells

Stuart et al. (2023) collected bone marrow mononuclear cells (BMMCs) from healthy human donors, and jointly profiled H3K27ac and H3K27me3 using scNTT-seq technology. We downloaded the processed R object from Zenodo (https://zenodo.org/record/7102159), which contains *n* = 5236 cells with top 71 253 bins from H3K27ac modality and top 43 170 bins from H3K27me3 (bin size =1 kb) used for the original analysis, where 15 different cell types were identified through WNN workflow on aggregated bin data by the authors. For our analysis, we obtained the genome bin features from fragment files and selected the top 25 000 bins (bin size =10 kb) using the same strategy described above.

#### 2.6.3 Human peripheral blood mononuclear cells

The peripheral blood mononuclear cell (PBMC) sample was obtained from a healthy female donor (*n* = 11 909 before quality control). The dataset was generated by 10× Genomics using single-cell multiome ATAC + Gene Expression (publicly available on 10× Genomics website). For each cell, 36 601 genes and 106 056 peaks were profiled in parallel. We followed the WNN workflow (Seurat V4) to generate the cell type labels through joint analysis of the transcriptomics (RNA-seq) and chromatin accessibility (ATAC-seq) profiles. We kept the cells (*n* = 11 402) that meet the specified criteria for quality control (number of ATAC-seq counts ∈[5000,70 000]; the number of RNA-seq counts ∈[1000,25 000],>20% mitochondrial counts). Top 5000 genes and top 25 460 peaks were selected for WNN analysis. As a result, the Louvain algorithm (resolution =0.25) led to 15 clusters, which were further used for method benchmark after cell type annotation. For ATAC peak data, the read (fragment end) count are converted to the fragment count, as suggested by Martens *et al.* (2024) (estimated fragment count=(odd read count+1)/2).

#### 2.6.4 Mouse cortex and hippocampus

Yao *et al.* (2021) sequenced approximately 1.3 million cells in the adult mouse cortex and hippocampus regions and obtained their transcriptomic profiles, leading to a thorough assortment of glutamatergic and GABAergic neuron types. For this study, we used the single-cell transcriptomic data generated by 10× Genomics Chromium platform (version 2 chemistry). We downloaded the processed data (*n* = 1 169 213) from the Neuroscience Multi-omic (NeMO) Data Archive as part of the BRAIN Initiative Cell Census Network. Out of genes measured in total, we selected 5000 highly variable genes from the normalized dataset using LIGER pipeline (Welch *et al.* 2019, Gao *et al.* 2021, Lu and Welch 2022). For evaluation on the investigated methods, we used 42 cell classes and subclasses annotated by the authors following Tasic *et al.*’s (2018) work.

#### 2.6.5 Mouse organogenesis

Argelaguet *et al.* (2022) investigated the mouse early organogenesis by simultaneously profiling gene expression and chromatin accessibility in the same nuclei (10× multiome) from mouse embryos between 7.5 and 8.75 days (E7.5–E8.75) of gastrulation. Specifically, we selected the E7.5, E8, E8.5, and E8.75 embryos ATAC-seq datasets (*n* = 68 804), and preprocessed the fragment files following the ArchR pipeline provided by the authors (Granja *et al.* 2021). After excluding the cells identified as low quality or doublets, we obtained the peak count matrix comprising 191 407 peaks from *n* = 41 705 cells. We further selected the 25 000 peak features with the highest total number of counts across all cells for analysis. Fragment count was estimated using read count following the same approach described above.

### 2.7 Experiments

We benchmarked the selected models through five-fold cross-validation over a variety of datasets. For model training, we used a mini-batch size of 128, Adam optimizer (learning rate =0.001). Each FC layer has 128 hidden units. The architecture incorporated Batch Normalization and ReLU activation functions in the majority of layers, alongside a dropout rate of 0.2 to prevent overfitting. We performed the Louvain algorithm (*k*-NNs: *k* = 20) on the latent cell embeddings for clustering. The algorithm was run with five random starts unless stated otherwise. The cluster assignment with the best quality was recorded. For the juvenile mouse brain data, the dimension of the latent space *D* was set to 30 and all models underwent 300 epochs of training (Supplementary Fig. S3a–c). For the BMMCs data, we used *D* = 30 and 200 training epochs. For single-cell unimodal datasets: *D* = 20 and 200 training epochs were employed for PBMCs data; *D* = 30 and 15 training epochs for mouse cortex and hippocampus data; *D* = 30 and 50 training epochs for mouse organogenesis data.

### 2.8 Model implementation

All reported VAE models were implemented in Pytorch 1.10.1 and Python 3.8, trained with 2.9 GHz Intel Xeon Gold 6226R and NVIDIA A40 GPU.

## 3 Results

### 3.1 1D CNNs for single-cell multimodal epigenomics integration

We introduce ConvNet-VAE, a novel approach designed to efficiently learn biologically meaningful low-dimensional cell representations from high-throughput single-cell multimodal epigenomic data. This framework capitalizes on recent advancements in chromatin profiling technologies which permit parallel measurements of histone modifications (e.g. H3K27ac, H3K27me3) and chromatin states at single-cell resolution (Bartosovic and Castelo-Branco 2023, Stuart *et al.* 2023). The sequenced fragments over the genome are obtained from each individual cell (Fig. 1a).

Because single-cell multimodal epigenomic experiments measure different features over the same sequential domain (i.e. the genome), we reasoned that the data are most naturally represented as a multichannel 1D sequential signal. This is a quite different approach than previous single-cell multimodal neural networks, which treat each modality as if it measured completely unrelated features (e.g. distinct genes or peak locations for each modality). Additionally, previous approaches often use a separate encoder and decoder network for each modality, while ours uses a single encoder and a single decoder that operate on multichannel signals. By operating on this multichannel representation of the data, we introduce an appropriate inductive bias that significantly reduces the number of parameters and enforces statistical dependence among neighboring genomic locations within a modality and across modalities at a given genomic locus.

ConvNet-VAE is a convolutional VAE based upon a Bayesian generative model (Fig. 1b). To apply 1D convolutional filters (Conv1D), the input multimodal data are transformed into 3D arrays (cell × modality × bin), following window-based genome binning at 10 kb resolution (Chen *et al.* 2019) (Fig. 1a). The encoder efficiently extracts latent factors, which are then mapped back to the input feature space by the decoder network. We use a discrete data likelihood (Poisson distribution) to directly model the observed raw counts.

We also extended ConvNet-VAEs to incorporate conditional information such as experimental batches, allowing batch correction using conditional VAEs, which has proven an effective strategy for scRNA-seq data (Lopez *et al.* 2018). In our model, the categorical variables (e.g. batch information) are one-hot encoded and then concatenated with the flattened convolutional layer outputs, instead of being combined directly with the multimodal fragment count data over the sorted genomic bins. We incorporated the conditional information in this way because, unlike fully connected layers, convolutional layers most naturally accommodate sequential data rather than one-hot encodings. Thus, we found it more natural to inject the batch information after the convolutional layers.

In the following sections, we showcase the effectiveness and superiority of ConvNet-VAEs by evaluating them on real data and comparing them with FC-VAEs.

### 3.2 ConvNet-VAEs learn cell representations using fewer parameters than FC-VAEs

A key advantage of ConvNet-VAEs is the proper inductive bias induced by convolution, which should result in considerable parameter savings. This advantage should increase with the number of modalities per cell: The number of peaks per modality usually exceeds 10^5^, and the peaks often do not overlap across modalities.

To investigate the advantage of ConvNet-VAEs on real data, we analyzed a recently published single-cell bi-modal dataset from juvenile mouse brains generated by the nano-CT technology (Bartosovic and Castelo-Branco 2022). After preprocessing, the dataset consists of 11 981 cells from four experimental batches with H3K27ac and H3K27me3 modalities. We extracted the top 25 000 bins identified across both modalities as the input feature set. We then separately examined the effects of (i) kernel size and stride and (ii) number of convolutional layers on the number of parameters and performance of ConvNet-VAE models. When examining the effects of kernel size and stride, we used architectures with a single convolutional layer and varied kernel size (K) and stride (S) from 11 to 51, with *K* = *S* in each case. Second, we examined the effects of varying the number of convolutional layers from 1 to 3, while keeping a fixed kernel size of 11 and stride of 3. (Note that we used a smaller *S* = 3 with multiple convolutional layers to avoid the output dimensionality being too small.) In the single layer setting, the receptive field is the same as the kernel size. For example, the kernel of size 51 covers 51 bins, equivalent to a genomic region of 510 kb, at each time. Given three layers (*K* = 11, *S* = 3), the receptive field is 131 bins (1310 kb). We compared all models against FC-VAEs. To ensure a fair comparison, we ran all models through five-fold cross-validation, with 300 training epochs.

Single-Conv1D-layer ConvNet-VAEs do indeed require fewer trainable parameters than FC-VAEs in this setting. For example, ConvNet-VAE (K51, S51) only uses 20% of the parameters that are needed for FC-VAEs, while ConvNet-VAE (K31, S31) uses 33%. As shown in Fig. 2c, as the number of convolutional layers increases, ConvNet-VAEs use fewer parameters. According to the UMAP visualization (colored by the published labels) of the cell embeddings obtained by the selected models, ConvNet-VAEs with varying *K*, *S*, and the number of layers result in qualitatively similar embeddings compared to the FC-VAE (Fig. 2a).

ConvNet-VAEs took slightly longer to complete the training (Fig. 2d), and the runtime increases nearly linearly as the number of modalities grows (Supplementary Fig. S3d and e). The most compact ConvNet-VAE (K51, S51) led to a 2.5% decrease in average marginal log-likelihood on the validation sets (Fig. 2e), but the (K11, S11) model achieved comparable or better marginal likelihood using 1M fewer parameters than the FC-VAE. Increasing the number of convolutional layers or stride resulted in a worse marginal likelihood. However, the models with slightly worse marginal likelihood still excelled in learning low-dimensional cell representations that could reproduce the published cluster assignments (Fig. 2f). The ARI first increased as more cell clusters were identified by the Louvain algorithm at a higher clustering resolution, then decreased due to potential over-clustering. ConvNet-VAE (K51, S51) achieved the same highest ARI of 0.83 (averaged over five random runs of the Louvain clustering) as single-layer FC-VAE did on the training sets, and beat FC-VAEs with ARI of 0.82(±0.01). Similarly, configured ConvNet-VAE with smaller kernels and stride displayed a comparable pattern in cluster counts and ARI scores. The two-layer ConvNet-VAE performed slightly better in terms of ARI than the one-layer ConvNet-VAE, while one fully connected layer performed the best, with each additional layer leading to a decline in performance. In summary, this first set of tests indicates that ConvNet-VAE can achieve similar or better performance compared with FC-VAE using fewer parameters.

### 3.3 ConvNet-VAEs show a larger advantage with increasing number of modalities per cell

Because our approach treats each modality as a different channel along a shared sequential domain, we expect the advantage of our approach to increase with the number of modalities profiled per cell. To investigate this, we expanded the analysis by incorporating a third modality, chromatin accessibility, which was measured alongside H3K27ac and H3K27me3 by the developers of nano-CT using ATAC-seq (Bartosovic and Castelo-Branco 2022). A total of 4434 cells from two experimental batches have ATAC, H3K27ac, and H3K27me3 profiles (three modalities per cell). As in the previous section, we selected the 25 000 bins with the highest counts across modalities and generated a 4434×3×25 000 input for ConvNet-VAEs.

**Figure 3.**
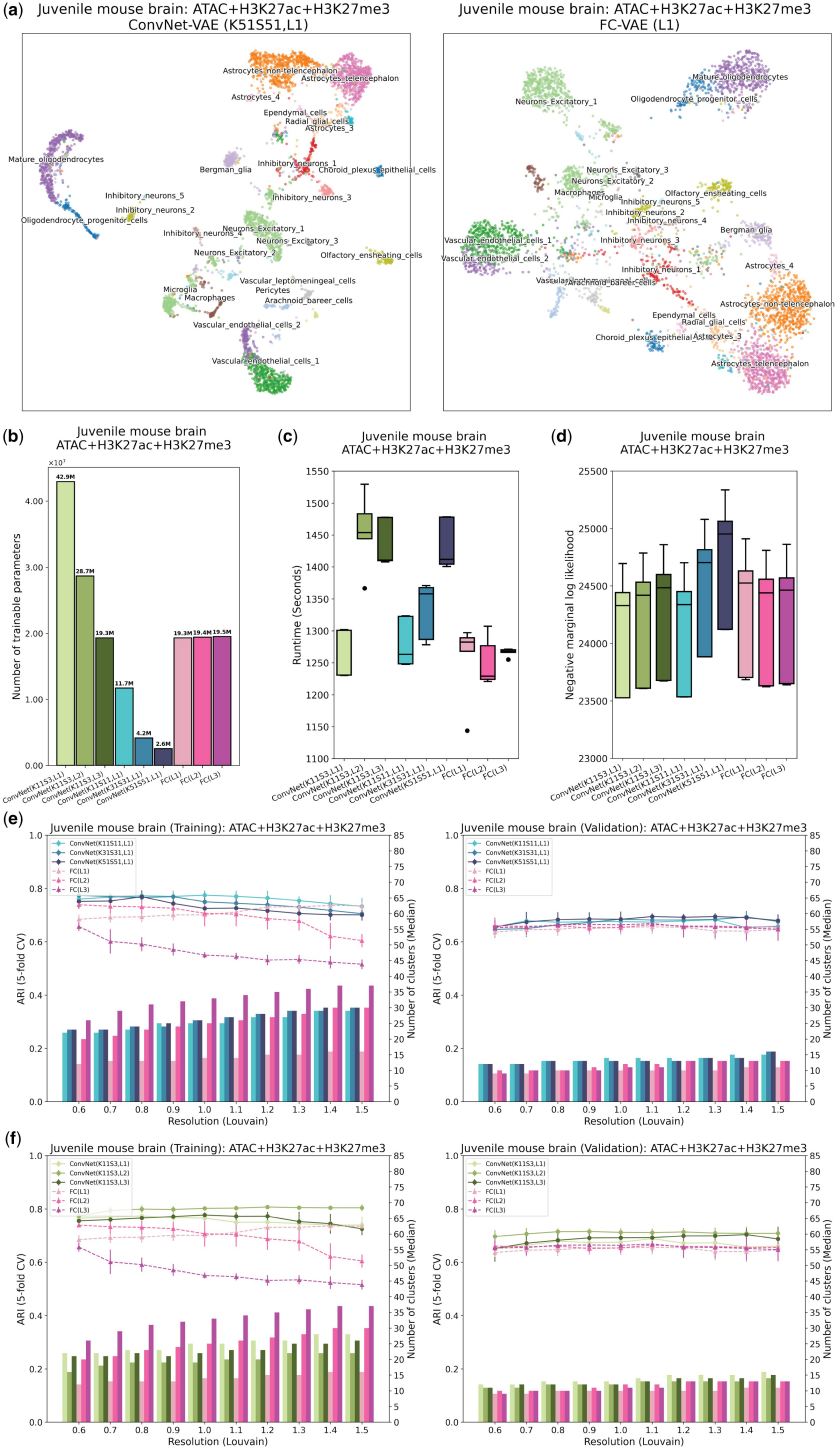
ConvNet-VAEs integrate single-cell tri-modal epigenomic profiling data from mouse brain. (a) UMAP visualization of cell embeddings from ConvNet-VAEs (left) and FC-VAE (right). (b) The number of trainable parameters of ConvNet-VAEs from Group 1, Group 2, and FC-VAEs. (c) Training time across five-fold cross-validation is reported for each model. (d) Negative marginal log-likelihood of validation set estimated through importance sampling (five-fold cross-validation). A lower value implies a larger marginal log-likelihood. (e, f) Comparisons between ConvNet-VAEs and FC-VAEs in terms of the quality of cell embeddings (training set: left; validation set: right). The bars show the median number of clusters obtained by the Louvain algorithm from five splits in cross-validation over a range of resolutions. The corresponding average Adjusted Rand Index (ARI) is calculated by comparing the resulting clusters to the published cell type labels, displayed as a line plot. Error bars indicate SD across five-fold cross-validation.

Through qualitative evaluation in the UMAP space, the single-Conv1D-layer ConvNet-VAE model with a large kernel and stride (K51, S51) results in more compact cell clusters than single-layer FC-VAE (Fig. 3a), while requiring 87% fewer trainable parameters (Fig. 3b). This efficiency remained notable even with a smaller kernel and stride (K11, S11), with a 39% reduction in parameters. The gap in runtime between ConvNet-VAEs and FC-VAEs also becomes narrower. For instance, single-Conv1D-layer ConvNet-VAE (K51, S51) takes 14% more time than the single-layer FC-VAEs to finish 300 training epochs, a decrease from the 25% longer runtime seen in the bi-modal analysis (Fig. 3c). There was no statistical difference in the marginal log-likelihoods across all investigated VAE variants (Fig. 3d), implying equivalent capabilities in modeling the data distribution.

The advantage of ConvNet-VAEs becomes even more apparent when evaluating the quality of the cell embeddings (Fig. 3e and f). On both training and validation sets, the single-Conv1D-layer ConvNet-VAEs lead in clustering accuracy (highest ARI: 0.78(±0.02) at resolution 1.0 for training 0.69(±0.01) at resolution 1.1 for validation), as compared to the FC-VAEs’ highest ARI of 0.74(±0.01) at resolution 0.6 for training and 0.67(±0.03) at resolution 1.1 for validation (Fig. 3e). This superiority is further supported by the performance of the multi-Conv1D-layer VAEs, which are top performers at almost all clustering resolutions (Fig. 3f). For example, 2-Conv1D-layer ConvNet-VAE (K11, S3) stands out by producing an ARI of 0.81(±0.01) and 0.72(±0.01) on the training and validation sets, respectively. Interestingly, unlike FC-VAEs, where additional layers usually lead to lower quality of the cell latent factors in the training data, ConvNet-VAEs can actually benefit from extra convolutional layers (Figs 2g and 3f). Furthermore, based on the results from unimodal, bi-modal, and tri-modal analyses, the ConvNet-VAEs show their ability to adopt the information from all molecular modalities for learning cell embeddings. They are more effective than FC-VAEs as more modalities are included, especially when the added modalities are more biologically informative (e.g. H3K27me3 → H3K27ac + H3K27me3) (Supplementary Fig. S4b). We have also observed that the inductive bias in ConvNet-VAEs allows the models to efficiently learn useful local patterns, using a reduced number of parameters. Such models of less complexity also lead to better generalization on small datasets (Supplementary Fig. S5).

### 3.4 ConvNet-VAEs allow for improved data integration

**Figure 4.**
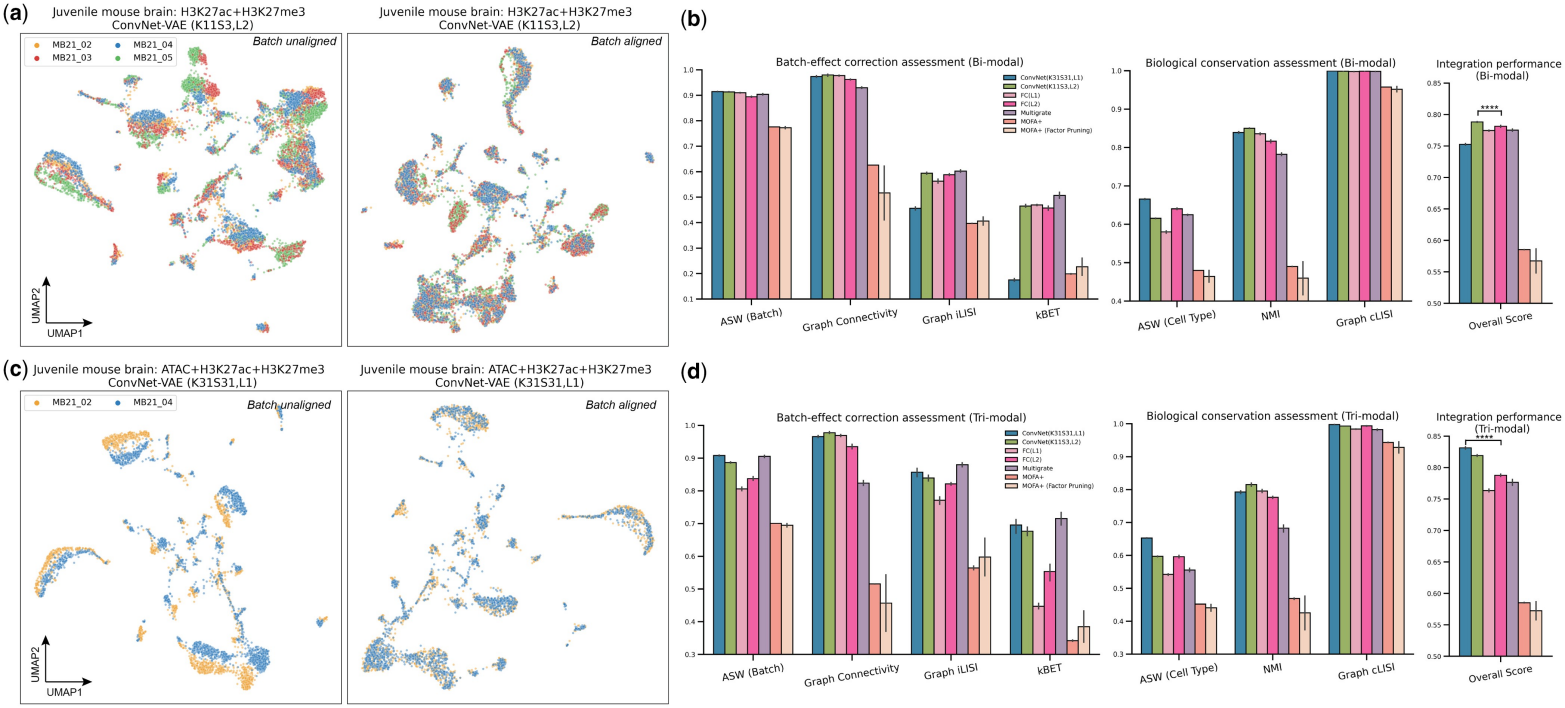
Benchmark of ConvNet-VAEs on data integration. VAE models were applied on the entire tested datasets without training/validation splitting. (a, c) UMAP visualizations of cell embeddings from selected models on the bi- and tri-modal data, before and after alignment. (b, d) Quantitative comparison of batch-effect correction (four metrics) and biological conservation (three metrics) among ConvNet-VAEs, FC-VAEs, Multigrate, and MOFA+. An overall score is calculated by taking an weighted average of these metrics (batch-effect correction and biological conservation scores are weighted at 0.4 and 0.6, respectively). The bars show average values with SD from five random runs. One-sided *t*-test is performed. Conventions for symbols indicating statistical significance: ${}^{****}P\leq .0001$.

Single-cell data are often generated from different experiments, leading to batch effects that stem from technical rather than biological differences. Therefore, correcting for these effects is essential for clustering and visualization to accurately reflect the underlying biology. A number of methods have been introduced to address this problem in single-cell uni-modal data (Korsunsky *et al.* 2019, Stuart *et al.* 2019, Welch *et al.* 2019, Gao *et al.* 2021). The same challenge occurs with these single-cell multimodal epigenomics datasets (Fig. 4c). Without removing batch effects, the cells with bi-modal and tri-modal measurements from different datasets are poorly aligned, resulting in clusters that separate by dataset rather than underlying biological cell type.

Here, we selected ConvNet-VAEs with single and multiple Conv1D layers to demonstrate their capacity to remove batch-associated technical variation. There are four different batches in the single-cell bi-modal juvenile mouse brain data with measurement of H3K27ac and H3K27me3. When we apply the ConvNet-VAE with two convolutional layers (K11, S3) with batch information, the cells are well mixed in each cluster (Fig. 4a). In the tri-modal setting (simultaneous profiling of chromatin accessibility, H3K27ac, and H3K27me3), the cells from two replicates are clearly separated as shown. Based on the quality of batch mixing, the architecture with a single Conv1D-layer (K31, S31) successfully aligned these cells from both batches (Fig. 4c).

Beyond the qualitative evaluation, we further carried out a quantitative assessment of how well the models correct for batch effects and conserve biological variations, among the proposed ConvNet-VAEs, the baseline model FC-VAEs, and other reported methods. Similar to FC-VAEs, Multigrate employs PoE but uses a different decoder structure (Lotfollahi *et al.* 2022). MOFA+ is a stochastic variational inference framework learning meaningful low-dimensional factors from single-cell multimodal data. We applied MOFA+ (GPU version) with and without its factor pruning functionality.

For the benchmark analysis, we calculated seven metrics: ASW (Batch), graph connectivity, graph iLISI, and kBET for batch-effect correction, as well as ASW (Cell type), NMI, and graph cLISI for biological conservation (Luecken *et al.* 2022). In comparison to FC-VAEs, both selected ConvNet-VAEs showed similar or better performance in terms of ASW (Batch) and Graph connectivity, while the ConvNet-VAE with a single convolutional layer (K31, S31) is less favored with respect to Graph iLISI and kBET. After taking into account the conservation of biological information, the two-layer ConvNet-VAE tops with a mean overall score of 0.788(±0.001), beating two-layer FC-VAE, the second best performer (P<5E−5) (Fig. 4b). Encouragingly, the ConvNet-VAEs excelled across almost all metrics as compared to the FC-VAEs in dealing with three modalities. The improvements in ASW (Batch) and kBET were particularly significant. The ConvNet-VAEs lead the overall integration score by a large margin, with the single-layer version being the best (P<2.5E−9) (Fig. 4d). In general, Multigrate performs well in correcting the batch effect; however, it gets lower biological conservation scores (i.e. ASW (Cell type) and NMI). MOFA+ trails behind in this data integration assessment.

### 3.5 ConvNet-VAEs integrate histone modifications from scNTT-seq data

In addition to nano-CT, Stuart *et al.* (2023) developed scNTT-seq, enabling genome-wide measurement of multiple histone modifications at single-cell resolution. In this part, we showcase the adaptability and consistent performance of ConvNet-VAEs when applied to multimodal data obtained through varied sequencing methods.

**Figure 5.**
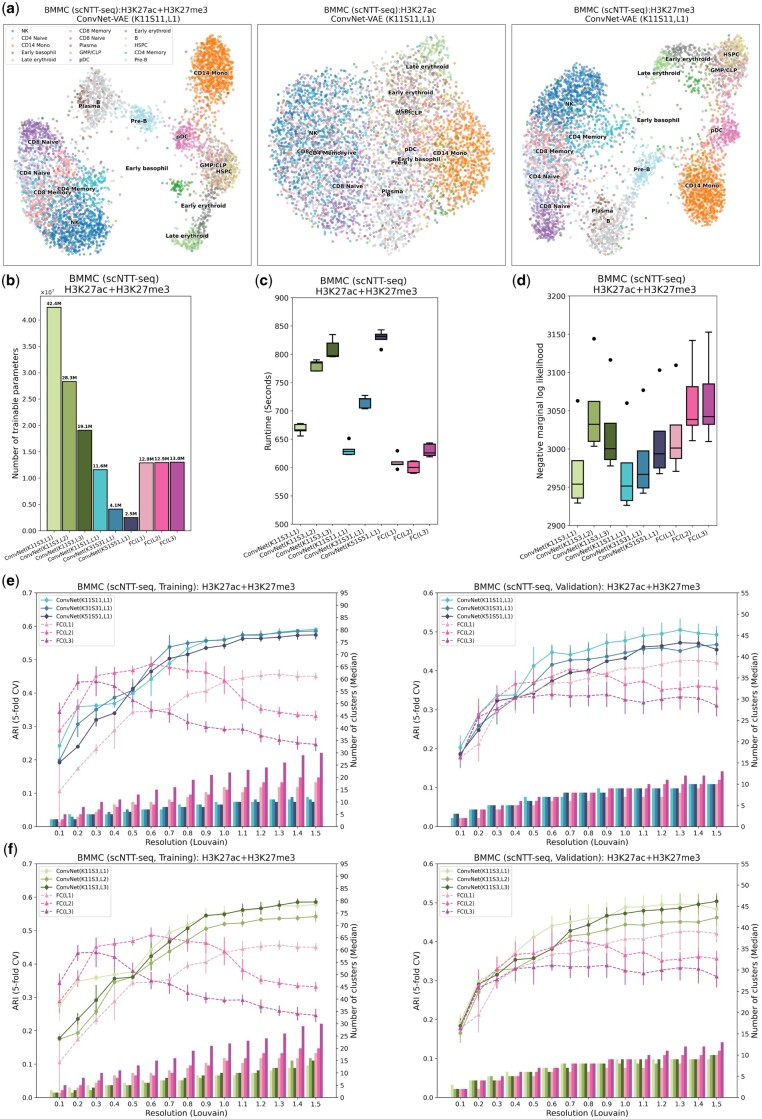
ConvNet-VAEs effectively integrate scNTT-seq data. (a) UMAP visualization of cell embeddings from ConvNet-VAE (single Conv1D layer, kernel size 11, stride 11) on BMMCs data containing H3K27ac and H3K27me3 (left), H3K27ac (middle), and H3K27me3 (right). (b) The number of trainable parameters of ConvNet-VAEs from Group 1, Group 2, and FC-VAEs. (c) Training time across five-fold cross-validation is reported for each model. (d) Negative marginal log-likelihood of validation set estimated through importance sampling (five-fold cross-validation). A lower value implies a larger marginal log-likelihood. (e, f) Comparisons between ConvNet-VAEs and FC-VAEs in terms of the quality of cell embeddings (training set: left; validation set: right). The bars show the median number of clusters obtained by the Louvain algorithm from five splits in cross-validation over a range of resolutions. The corresponding average Adjusted Rand Index (ARI) is calculated by comparing the resulting clusters to the published cell type labels, displayed as a line plot. Error bars indicate SD across five-fold cross-validation.

Toward this goal, we integrated single-cell bi-modal (H3K27ac and H3K27me3) epigenomic data profiled from BMMCs of healthy human donors (*n* = 5236). According to the UMAP plots, H3K27ac itself does not carry sufficient information to distinguish different cell types, whereas H3K27me3 provides sufficient information to identify the major cell types. Combining both modalities with the selected single-layer ConvNet-VAE (K11, S11), we achieved more compact cell clusters (Fig. 5a).

Although training ConvNet-VAEs with multiple convolutional layers, or those with larger kernels and strides, might require additional time compared to FC-VAEs (Fig. 5c), the ConvNet-VAEs display comparable or superior performance in estimating the marginal log-likelihood for the validation data, making them preferable as generative models (Fig. 5d). More strikingly, the proposed ConvNet-VAEs outperform the FC-VAEs on the training and validation sets by a large margin, when comparing ARI as the measure of the effectiveness of dimension reduction (Fig. 5e and f). Further, ConvNet-VAEs leverage cross-modal information rather than disregarding the less informative modality, aligning with our previous findings (Supplementary Fig. S4a).

In order to examine whether the convolutional layers are able to exploit the sequential relationships among genomic locations, we randomly shuffled genomic bins from this BMMC dataset and reanalyzed it with ConvNet-VAEs. The decline in ARI upon bin shuffling confirmed that the convolutional layers are indeed sensitive to local epigenomic patterns. In terms of the marginal log-likelihood, the negative effect brought by bin shuffling becomes more apparent when a larger kernel size is used (Supplementary Fig. S10). All these observations underscore the ability of 1D convolutional layers to capture spatial dependencies in the tested single-cell multimodal epigenomic data.

Moreover, we investigated the applicability of ConvNet-VAEs on unimodal single-cell data. In analyses of PBMC gene expression, PBMC ATAC (peaks), as well as the mouse organogenesis ATAC (peaks), single-Conv1D-layer ConvNet-VAEs perform on par with FC-VAEs. ConvNet-VAEs lead the performance in reducing the dimension of the large-scale mouse cortex and hippocampus transcriptomic profile (Supplementary Figs S11–S14). We also benchmarked ConvNet-VAEs against ArchR (Granja *et al.* 2021) and the latest SnapATAC2 (Zhang *et al.* 2023) frameworks using an scATAC-seq dataset generated for studying human hematopoietic differentiation (Buenrostro *et al.* 2018). This dataset comes with the ground truth labels obtained from FACS sorting. While SnapATAC2 and ArchR run quite fast, the quality of cell embeddings is far worse than that obtained by the ConvNet-VAE (Supplementary Fig. S1). For the RNA modality, we observed that using gene expression directly as input yielded better results compared to implementing the genomic binning strategy.

## 4 Discussion

In this study, we proposed the ConvNet-VAE framework, specifically designed to model single-cell multimodal epigenomic data. This model comprises 1D convolutional layers and hence takes multichannel binned fragment counts as input. The encoder network within this framework learns low-dimensional representations of cells that facilitate cell type inference following clustering. We validated ConvNet-VAEs’ utilities through integrative analyses of bi-modal (H3K27ac + H3K27me3) and tri-modal juvenile mouse brain data (ATAC + H3K27ac + H3K27me3), as well as bi-modal data from human bone marrow mononuclear cells (H3K27ac + H3K27me3).

As demonstrated by the results, ConvNet-VAEs are able to extract information about chromatin states and histone modifications, accurately capture the data distribution, and correct for batch effects. The 1D convolution layers are capable of capturing the spatial relationships among sequentially arranged genomic bins, as well as the cross-modal information. In qualitative and quantitative benchmarking with FC-VAEs, which solely utilize FC layers, ConvNet-VAEs show effectiveness by achieving on-par or enhanced performance using far fewer parameters. Due to inductive bias, ConvNet-VAEs with fewer parameters are less prone to overfitting and can generalize more effectively when working with limited data. Unlike FC-VAEs, ConvNet-VAEs can also benefit from including more layers (Conv1D) in the model architecture. Notably, the advantage of ConvNet-VAEs over FC-VAEs becomes more evident when jointly analyzing multiple modalities.

Nevertheless, the ConvNet-VAEs presented in this report are not without limitations. Due to the use of convolutional filters, they require that all modalities share the same feature space (i.e. an identical set of bins). The presented architecture is not able to accommodate for missing modalities. In our study, the tracks from different chromosomes are concatenated in our proposed ConvNet-VAE framework, and the kernel filters may cover bins/peaks from two different chromosomes at the boundaries. Despite the concern of discontinuities, the biological and sequential information are properly preserved by the ConvNet-VAEs. Hence, there is potential to further refine model performance by optimizing parameters like kernel size and stride length. We can take additional steps to manage this issue of discontinuities. One option is to use small bin and kernel sizes. In this way, the model focuses on extracting very local patterns. Given the high-dimensional feature space, the information captured at the small number of boundary regions will be overwhelmed by that extracted from other regions. Another potential solution is to zero-pad these boundaries in the input, and maintain the padding to ensure that features near the boundaries remain separate after each convolutional layer.

To summarize, the ConvNet-VAE framework stands out for its performance in integrating single-cell multimodal epigenomic data. We anticipate that the utility of our approach will become more promising as the number of modalities and cells in single-cell multimodal epigenomic datasets increases in the future.

## Supplementary Material

btae705_Supplementary_Data

## Data Availability

The published raw datasets are listed below. The processed count data used for analyses can be accessed on figshare: https://figshare.com/s/e5696dd20f7b0603f122. Juvenile mouse brain from Bartosovic *et al.* (2023) (GSE198467). Human bone marrow mononuclear cells (BMMCs) from Stuart *et al.* (2023) (GSE212588). Human peripheral blood mononuclear cells from 10× Genomics (2021) (https://www.10xgenomics.com/resources/datasets). Mouse cortex and hippocampus from Yao *et al.* (2021) (https://data.nemoarchive.org/biccn/grant/u19_zeng/zeng/transcriptome/scell/10x_v2/mouse/processed/YaoHippo2020/). Mouse organogenesis from Argelaguet *et al.* (2022) (GSE205117). Human hematopoietic differentiation from Buenrostro *et al.* (2018) (https://github.com/kaizhang/single-cell-benchmark/blob/main/data.yaml)
