# Supplementary material for: Integrating single-cell multimodal epigenomic data using 1D convolutional neural networks
Source: Bioinformatics. 2025 Jan 16;41(1):btae705. doi: 10.1093/bioinformatics/btae705 (PMC11751632; doi:10.1093/bioinformatics/btae705)
Supplement: btae705_Supplementary_Data [file btae705_supplementary_data.zip › ConvNetVAE_manuscript_bioinfo_supplementary.pdf]

Supplementary Note: Integrating single-cell multimodal epigenomic data using 1D-convolutional neural networks

Chao Gao[1], Joshua D. Welch[1,2]

[1] Department of Computational Medicine and Bioinformatics, University of Michigan, Ann Arbor MI 48109, USA
[2] Department of Computer Science and Engineering, University of Michigan, Ann Arbor MI 48109, USA
gchao@umich.edu, welchjd@umich.edu

1 Supplementary table

Model	Louvain	Leiden
ConvNet-VAE(K31S31, L1)	**0.745 (±0.009)**	**0.721 (±0.020)**
SnapATAC2	0.566	0.658
ArchR	0.142 (±0.001)	0.176 (±0.001)

Table S1. Clustering accuracy (ARI) benchmark on human hematopoiesis data. ARI is calculated using the Louvain and Leiden methods over a range of clustering resolution (0.1-1.5) for all models. The best results from ConvNet-VAEs and ArchR (5 runs each) are presented as mean values (± standard deviation). The best result from a single run of SnapATAC2 is reported.

2 Supplementary figures

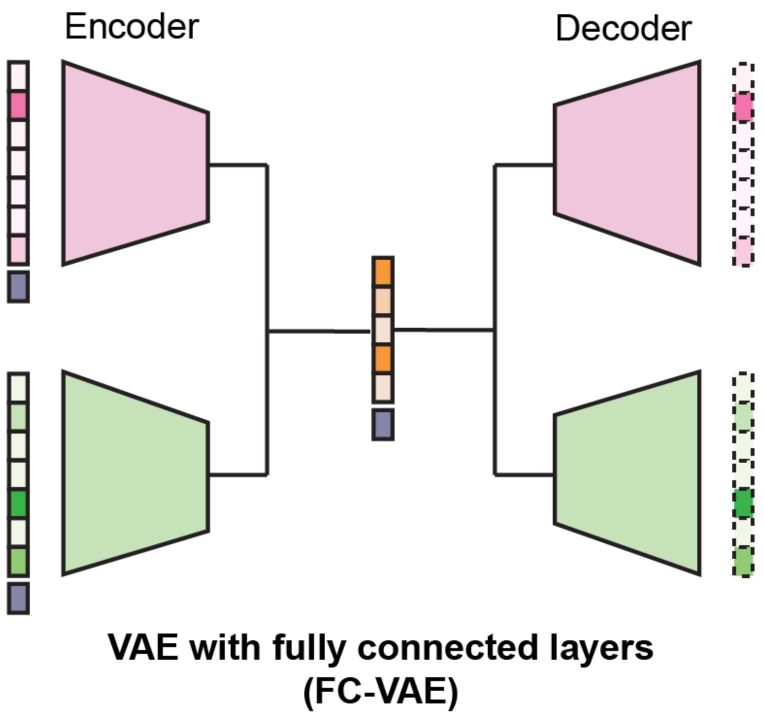

Fig. S1. Architecture of `FC-VAE` (bi-modal). A brief illustration of the architecture of a bi-modal `FC-VAE` based on Product of Experts (PoE). Each expert corresponds to a modality. It easily extends to additional modalities by adding encoder-decoder pairs.

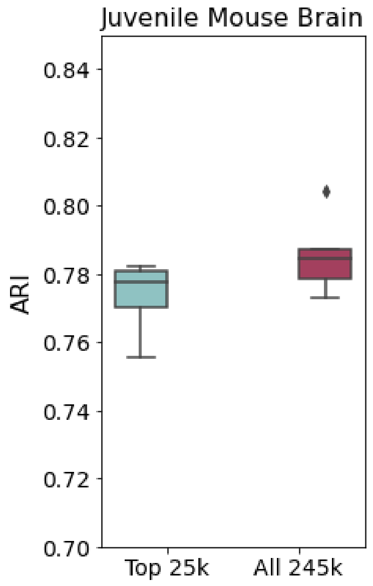

Fig. S2. Comparison of clustering accuracy across number of genomic bins utilized. The models are trained on juvenile mouse brain tri-modal dataset ($N = 4,434$). The results from 5 runs with random model initialization are reported.

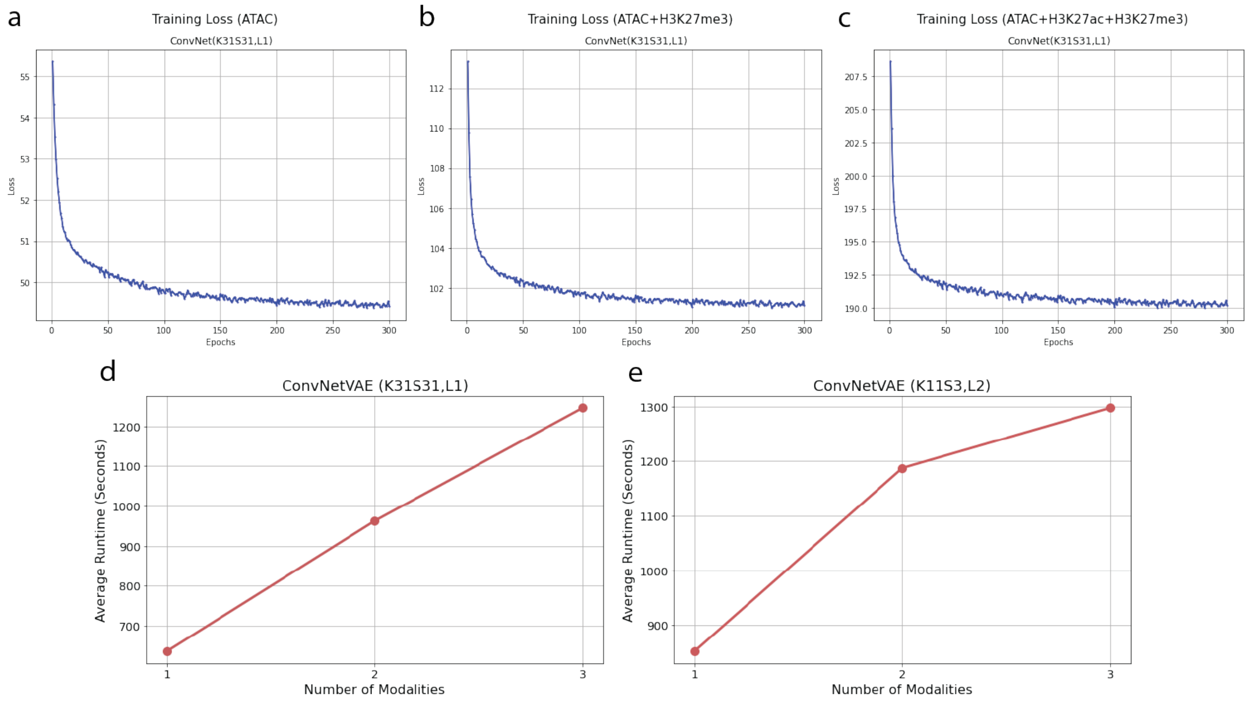

Fig. S3. Model convergence and runtime. The models are trained using 4,434 cells from the juvenile mouse brain dataset. (**a-c**) Training loss of `ConvNet-VAE` (K31S31, L1) on unimodal, bi-modal and tri-model data. (**d,e**) Average runtime required for 300 epochs of training.

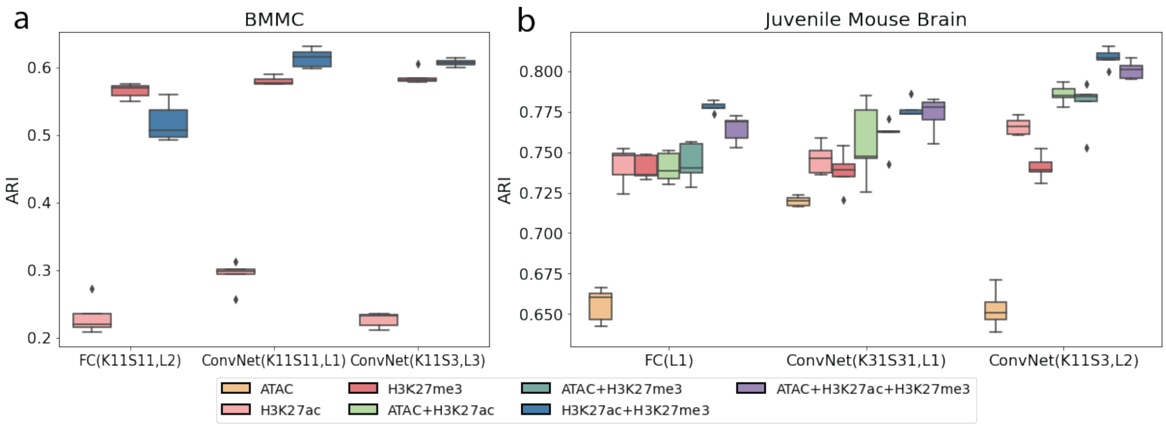

Fig. S4. Comparison of clustering accuracy across different combinations of modalities utilized. (**a**) The results from `FC-VAE`, `ConvNet-VAE`s (5 initializations each) on human BMMC data ($N = 11,981$). (**b**) The results from `FC-VAE`, `ConvNet-VAE`s (5 initializations each) on mouse juvenile mouse brain data ($N = 4,434$).

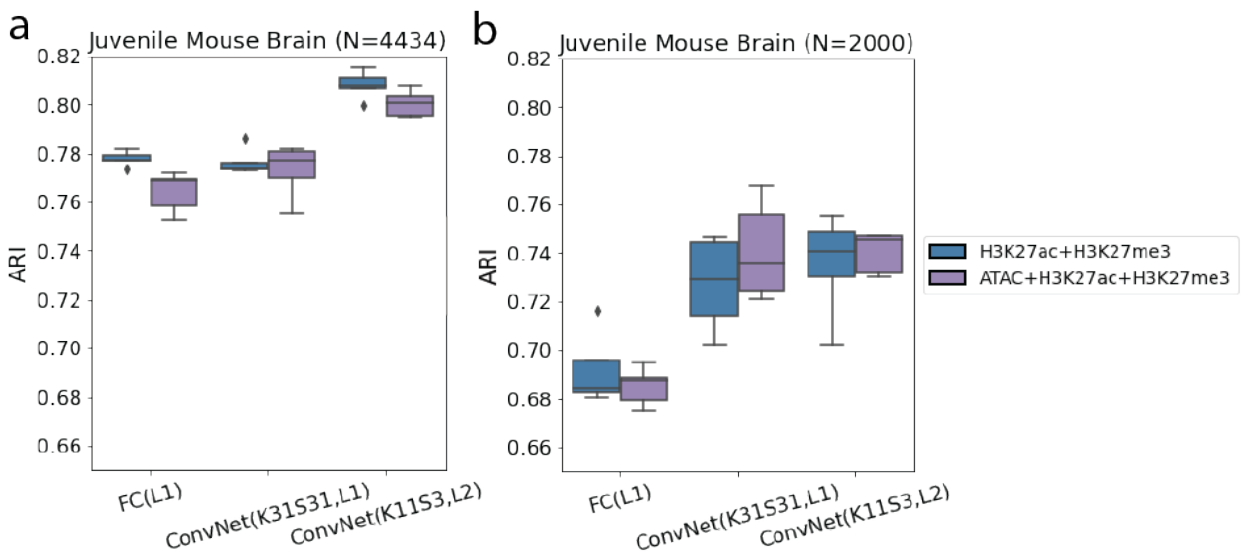

Fig. S5. Comparison of clustering accuracy across different numbers of cells utilized. The results from FC-VAE, ConvNet-VAEs (5 initializations each) on juvenile mouse brain data.

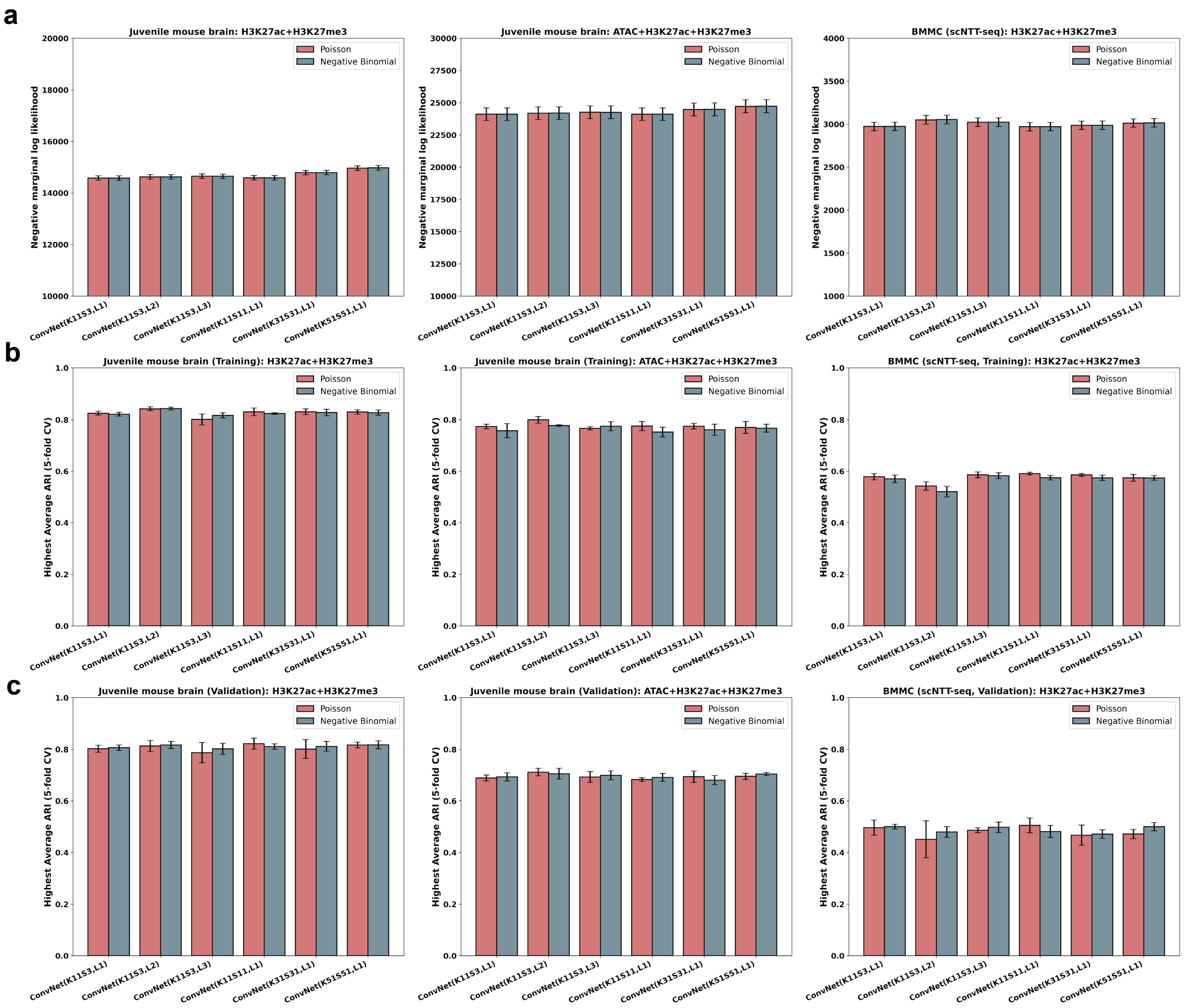

Fig. S6. Models with Poisson and negative binomial distributions lead to comparable performance on studied datasets. Bi-modal juvenile mouse brain (Left column), tri-modal juvenile mouse brain (Middle column), BMMCs (Right column). (**a**) Comparison of the marginal log likelihood (validation set) from `ConvNet-VAE`s under Poisson and negative binomial distributional assumption. (**b**) The highest average ARI that each model can achieve on the training sets over a range of clustering resolutions. (**c**) The highest average ARI that each model can achieve on the validation sets over a range of clustering resolutions. All error bars indicate the standard deviation from 5-fold cross-validation.

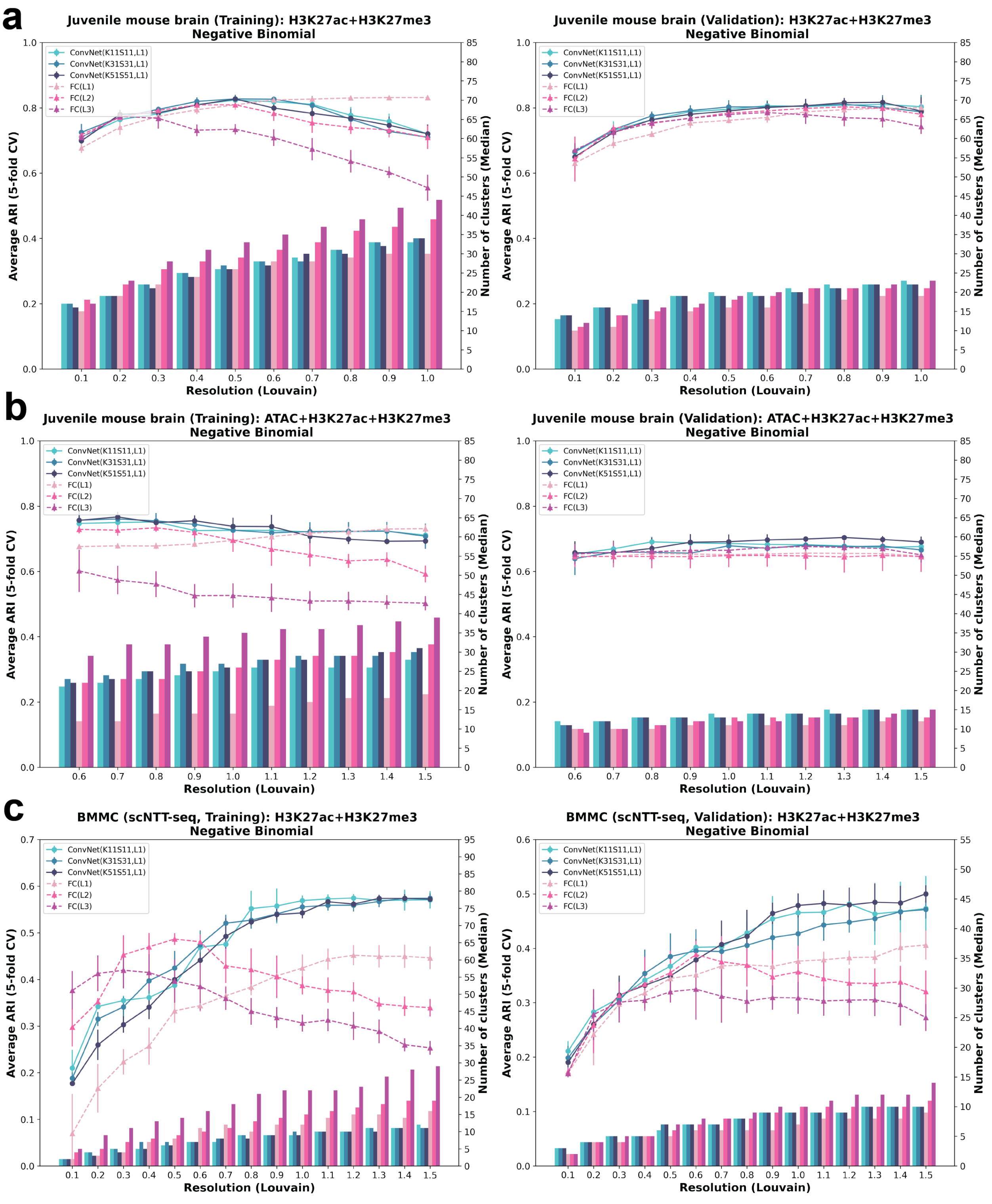

Fig. S7. Evaluation of 1-Conv1D-layer `ConvNet-VAE`s with negative binomial distribution: ARI. Comparison between `ConvNet-VAE`s (Group 1) using negative binomial modeling and `FC-VAE`s on the quality of cell embeddings, evaluated by ARI. (**a**) Bi-modal juvenile mouse brain. (**b**) Tri-modal juvenile mouse brain. (**c**) BMMCs. Error bars indicate the standard deviation from 5-fold cross-validation.

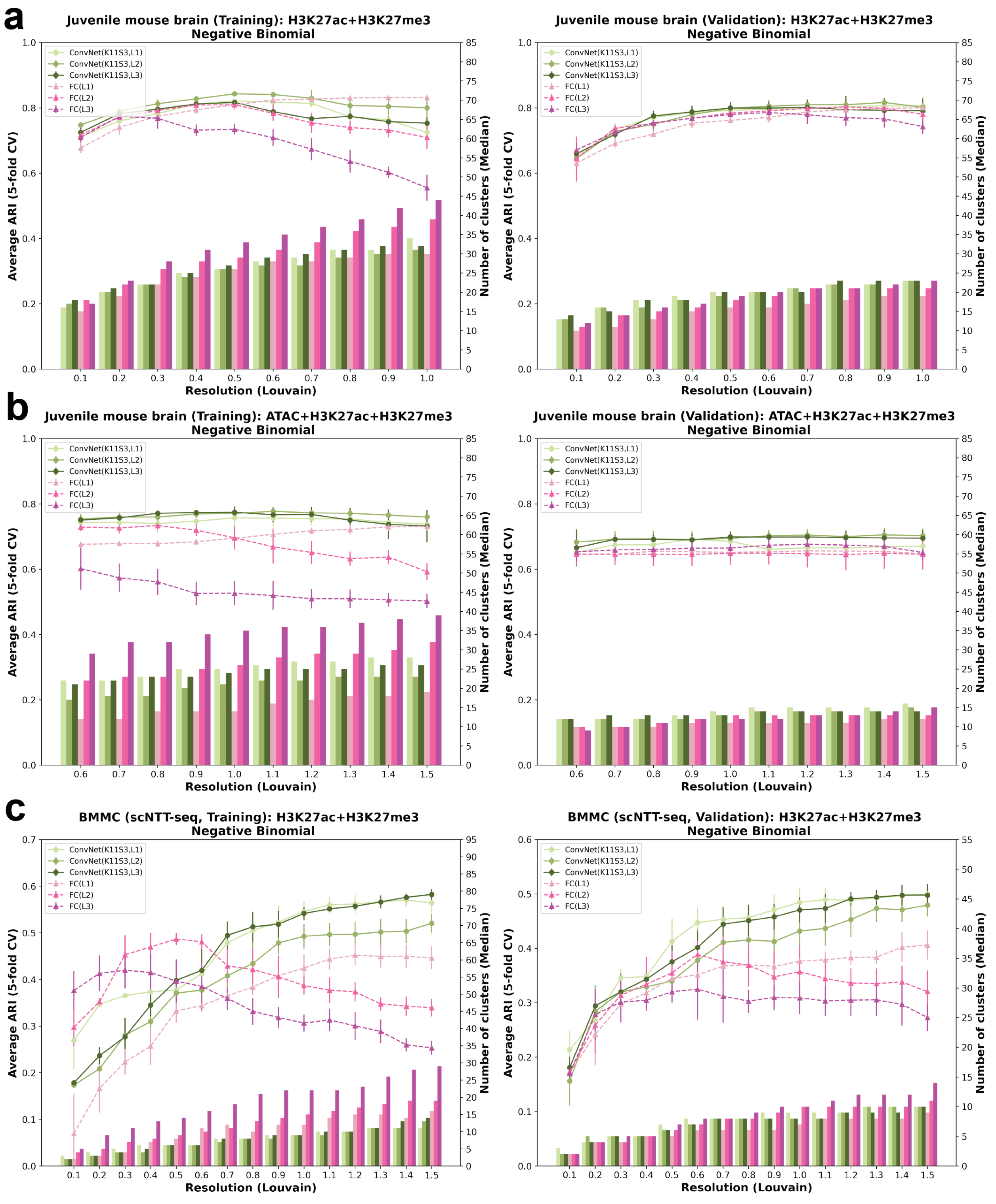

Fig. S8. Evaluation of multi-Conv1D-layer `ConvNet-VAE`s with negative binomial distribution: ARI. Comparison between `ConvNet-VAE`s (Group 2) using negative binomial modeling and `FC-VAE`s on the quality of cell embeddings, evaluated by ARI. (**a**) Bi-modal juvenile mouse brain. (**b**) Tri-modal juvenile mouse brain. (**c**) BMMCs. Error bars indicate the standard deviation from 5-fold cross-validation.

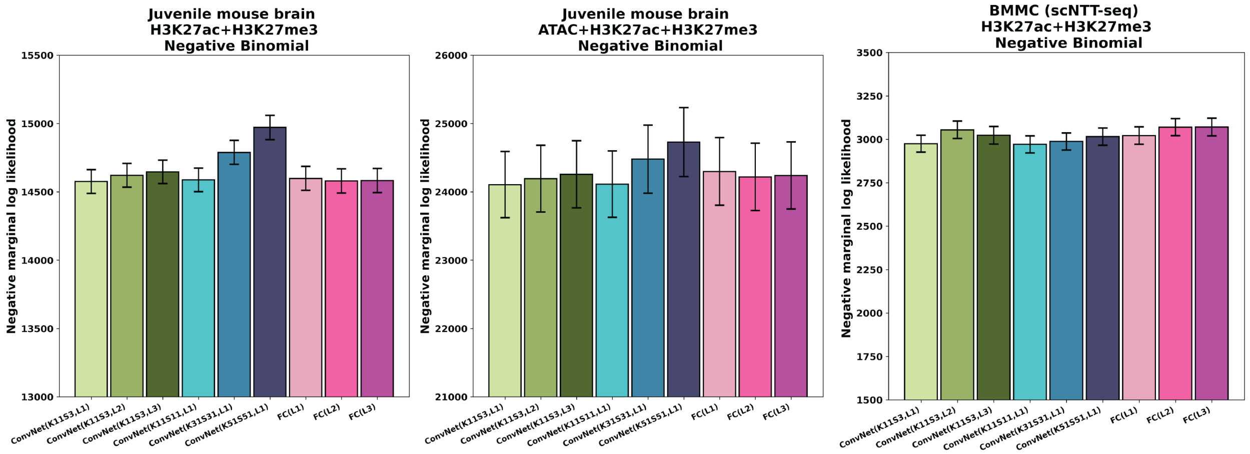

Fig. S9. Evaluation of `ConvNet-VAE`s with negative binomial: Marginal log likelihood (Validation). Comparison of the marginal log likelihood of the validation set between `ConvNet-VAE`s (negative binomial modeling) and `FC-VAE`s.

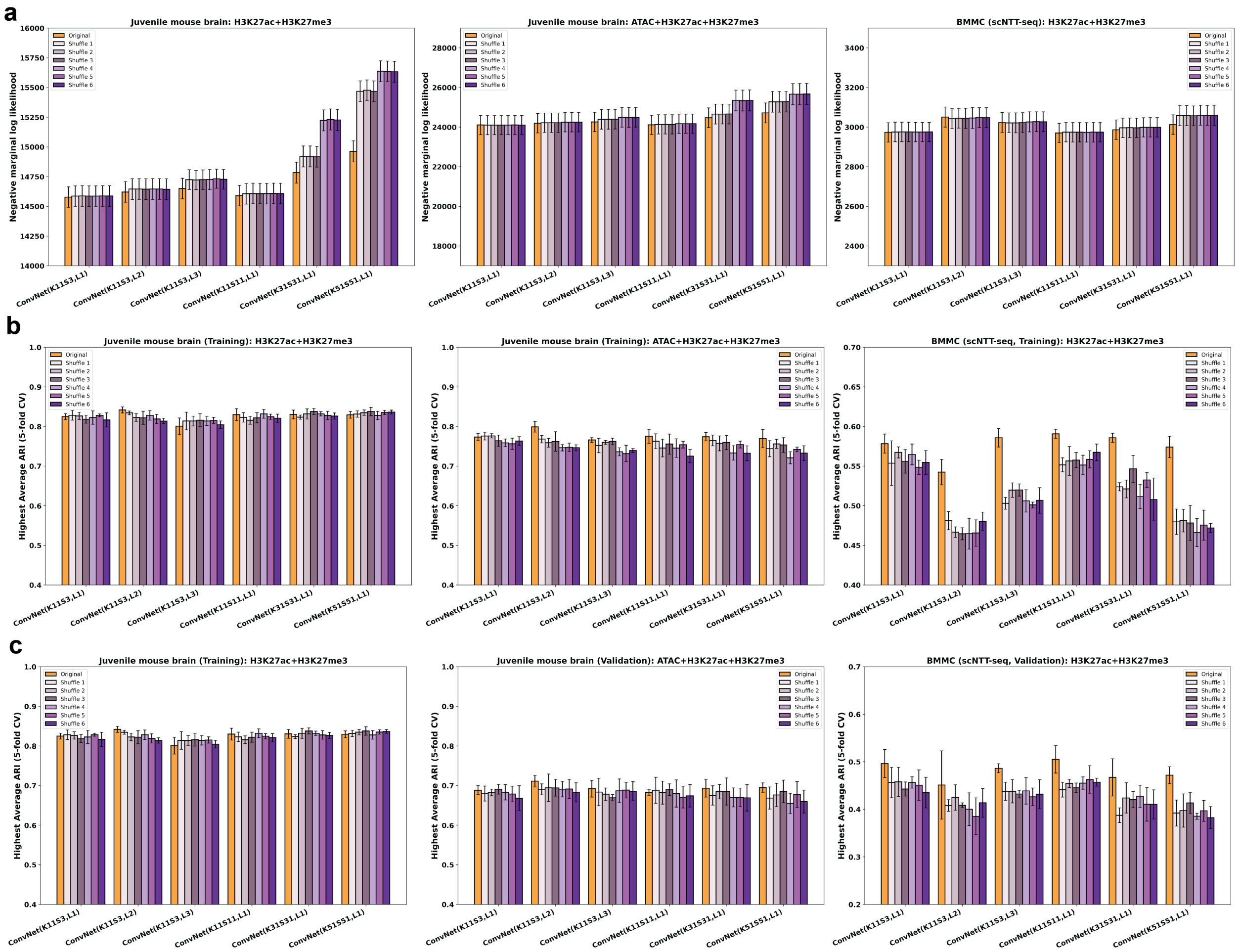

Fig. S10. Performance of `ConvNet-VAEs` after shuffling genomic bins. Side-by-side comparison between the results from the ordered bins and shuffled bins. Bi-modal juvenile mouse brain (Left column), tri-modal juvenile mouse brain (Middle column), BMMCs (Right column). (**a**) Comparison of the marginal log likelihood (validation set). (**b**) The highest average ARI that each model can achieve on the training sets over a range of clustering resolutions. (**c**) The highest average ARI that each model can achieve on the validation sets over a range of clustering resolutions. Error bars indicate the standard deviation from 5-fold cross-validation.

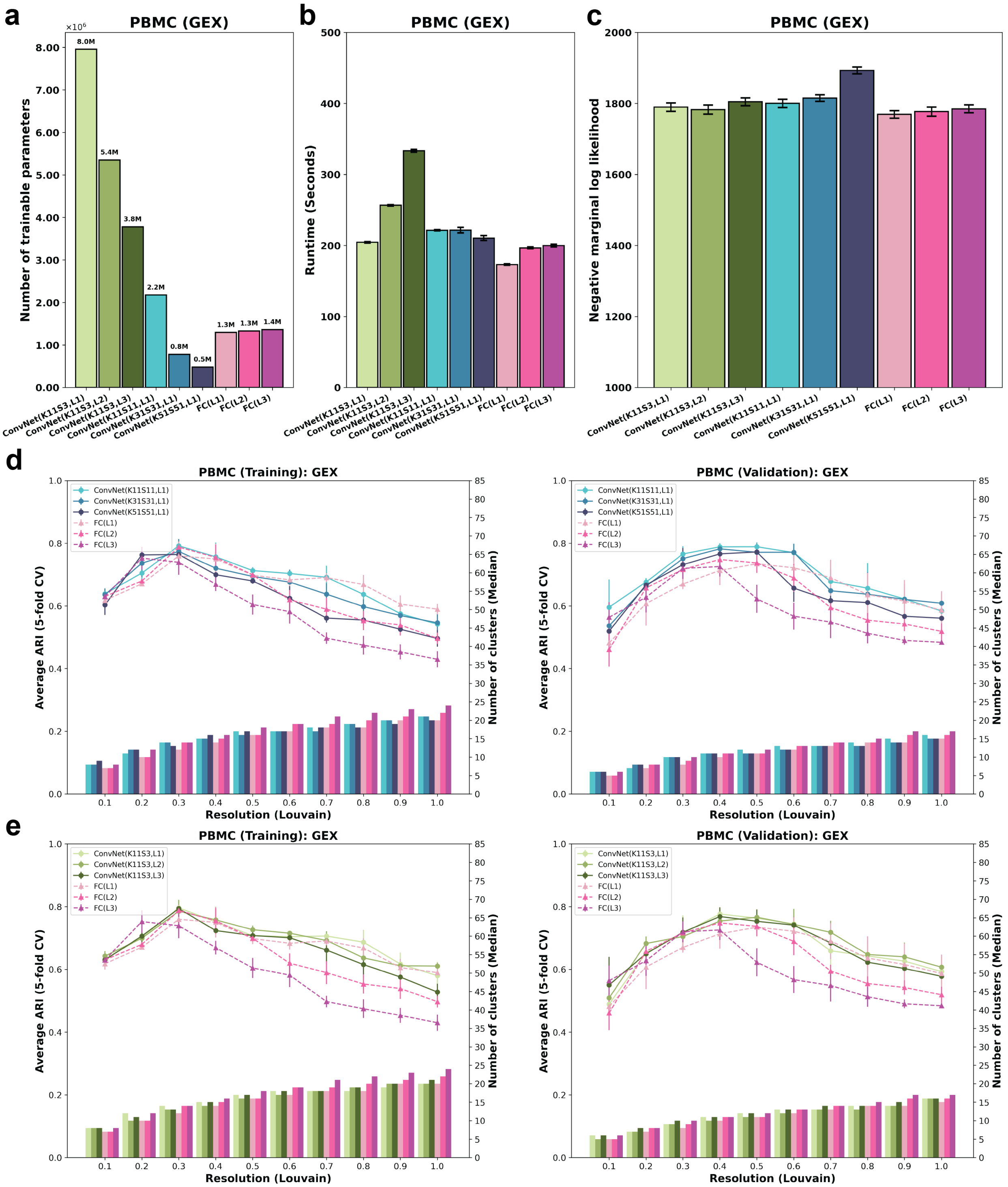

Fig. S11. Evaluation of `ConvNet-VAE`s on PBMCs (gene expression). (**a**) The number of trainable parameters of `ConvNet-VAE`s from Group 1 (Blue), Group 2 (Green), and `FC-VAE`s (Pink). (**b**) Average training time is reported for each model. Error bars indicate standard deviation across 5-fold cross-validation. (**c**) Average negative marginal log likelihood of validation set estimated through importance sampling. (**d,e**) Comparisons between `ConvNet-VAE`s and `FC-VAE`s on cell embeddings' quality. The bars show the median number of clusters obtained by the Louvain algorithm from 5 splits in cross-validation over a range of resolutions. The corresponding average Adjust Rand Index (ARI) is calculated by comparing the resulting clusters to the published cell type labels (line plot). Error bars indicate standard deviation across 5-fold cross-validation.

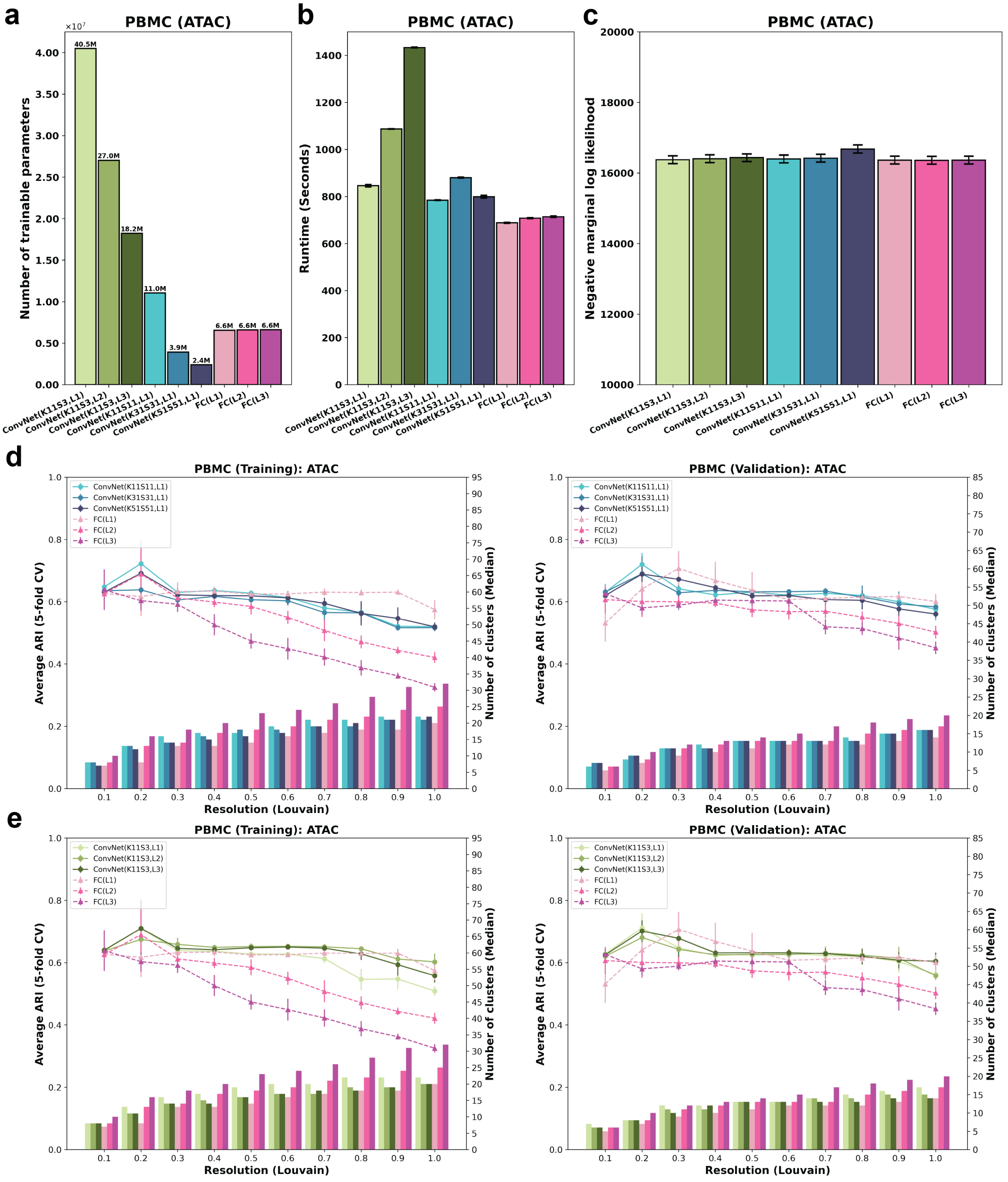

Fig. S12. Evaluation of `ConvNet-VAE`s on PBMCs (ATAC peaks). (**a**) The number of trainable parameters of `ConvNet-VAE`s from Group 1 (Blue), Group 2 (Green), and `FC-VAE`s (Pink). (**b**) Average training time is reported for each model. Error bars indicate standard deviation across 5-fold cross-validation. (**c**) Average negative marginal log likelihood of validation set estimated through importance sampling. (**d,e**) Comparisons between `ConvNet-VAE`s and `FC-VAE`s on cell embeddings' quality. The bars show the median number of clusters obtained by the Louvain algorithm from 5 splits in cross-validation over a range of resolutions. The corresponding average Adjust Rand Index (ARI) is calculated by comparing the resulting clusters to the published cell type labels (line plot). Error bars indicate standard deviation across 5-fold cross-validation.

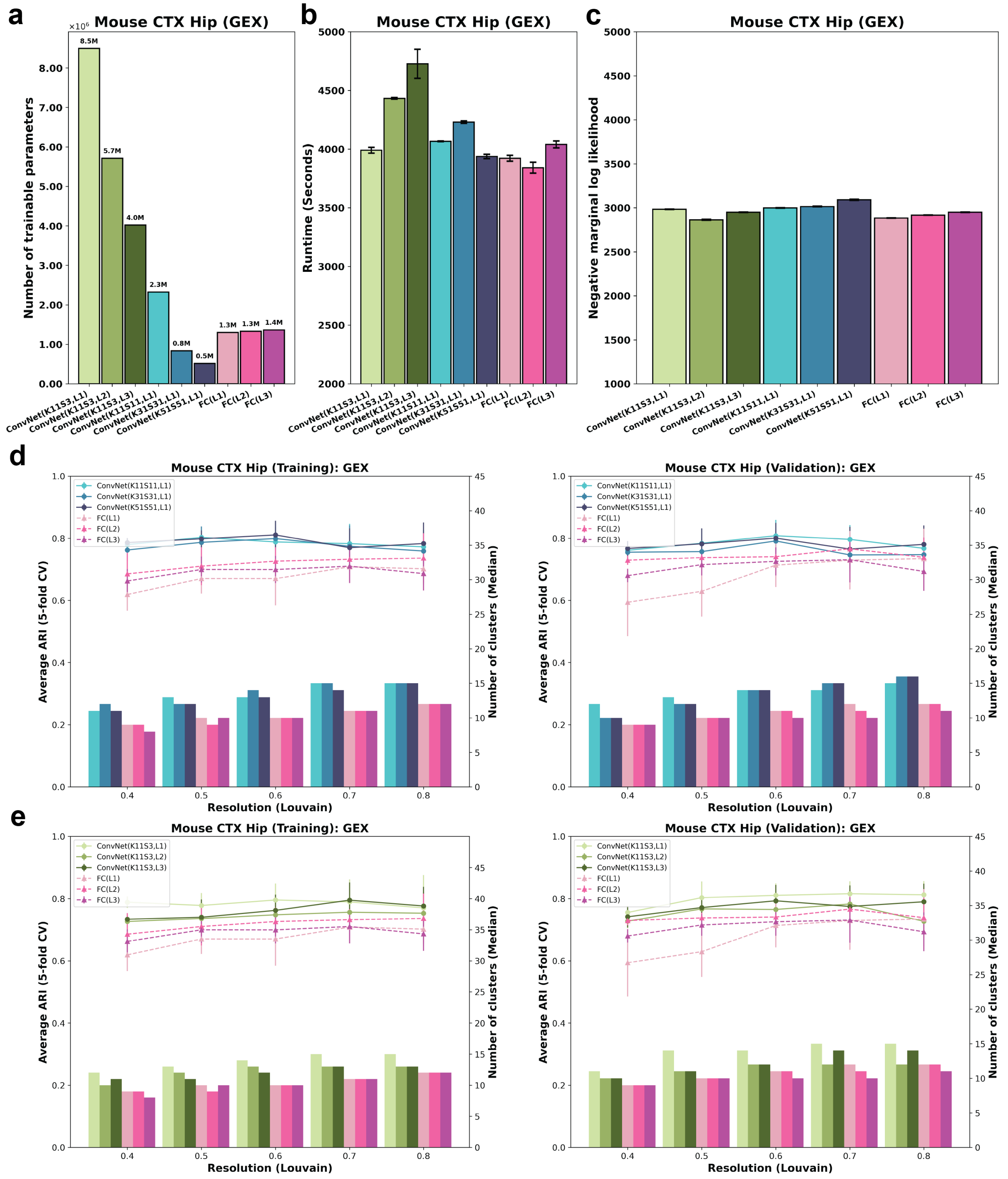

Fig. S13. Evaluation of `ConvNet-VAE`s on mouse cortex and hippocampus (gene expression). (**a**) The number of trainable parameters of `ConvNet-VAE`s from Group 1 (Blue), Group 2 (Green), and `FC-VAE`s (Pink). (**b**) Average training time is reported for each model. Error bars indicate standard deviation across 5-fold cross-validation. (**c**) Average negative marginal log likelihood of validation set estimated through importance sampling. (**d,e**) Comparisons between `ConvNet-VAE`s and `FC-VAE`s on cell embeddings' quality. The bars show the median number of clusters obtained by the Louvain algorithm from 5 splits in cross-validation over a range of resolutions. The corresponding average Adjust Rand Index (ARI) is calculated by comparing the resulting clusters to the published cell type labels (line plot). Error bars indicate standard deviation across 5-fold cross-validation.

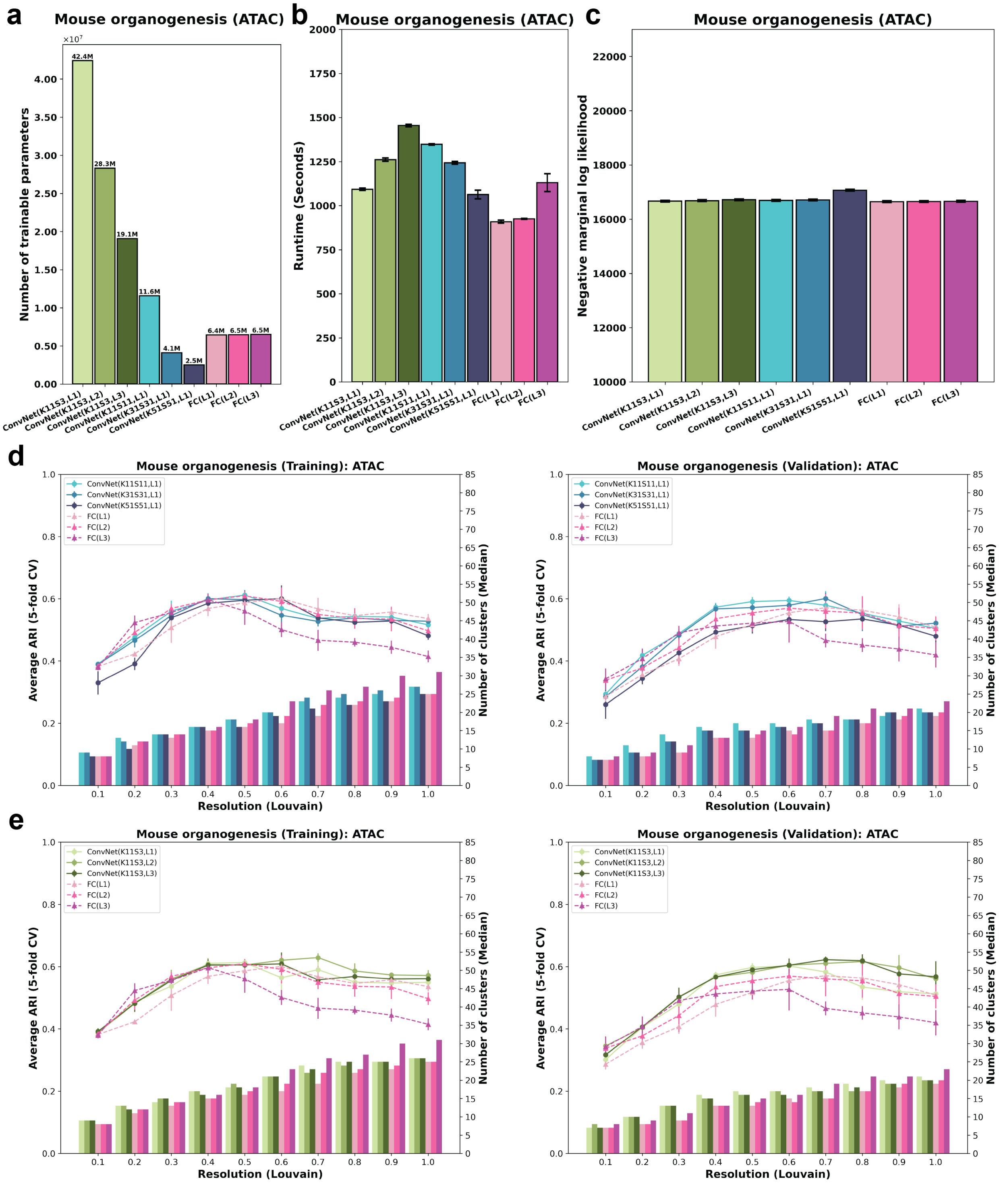

Fig. S14. Evaluation of ConvNet-VAEs on mouse organogenesis (ATAC peaks). (**a**) The number of trainable parameters of `ConvNet-VAEs` from Group 1 (Blue), Group 2 (Green), and `FC-VAEs` (Pink). (**b**) Average training time is reported for each model. Error bars indicate standard deviation across 5-fold cross-validation. (**c**) Average negative marginal log likelihood of validation set estimated through importance sampling. (**d,e**) Comparisons between `ConvNet-VAEs` and `FC-VAEs` on cell embeddings' quality. The bars show the median number of clusters obtained by the Louvain algorithm from 5 splits in cross-validation over a range of resolutions. The corresponding average Adjust Rand Index (ARI) is calculated by comparing the resulting clusters to the published cell type labels (line plot). Error bars indicate standard deviation across 5-fold cross-validation.
